# MicroRNA Expression Characterizes Oligometastasis(es)

**DOI:** 10.1371/journal.pone.0028650

**Published:** 2011-12-13

**Authors:** Yves A. Lussier, H. Rosie Xing, Joseph K. Salama, Nikolai N. Khodarev, Yong Huang, Qingbei Zhang, Sajid A. Khan, Xinan Yang, Michael D. Hasselle, Thomas E. Darga, Renuka Malik, Hanli Fan, Samantha Perakis, Matthew Filippo, Kimberly Corbin, Younghee Lee, Mitchell C. Posner, Steven J. Chmura, Samuel Hellman, Ralph R. Weichselbaum

**Affiliations:** 1 Comprehensive Cancer Center, University of Chicago, Chicago, Illinois, United States of America; 2 Ludwig Center for Metastasis Research, University of Chicago, Chicago, Illinois, United States of America; 3 Department of Medicine Center for Biomedical Informatics, University of Chicago, Chicago, Illinois, United States of America; 4 Institute for Genomics and Systems Biology, University of Chicago, Chicago, Illinois, United States of America; 5 Department of Radiation and Cellular Oncology, University of Chicago, Chicago, Illinois, United States of America; 6 Department of Pathology Committee on Cancer Biology, University of Chicago, Chicago, Illinois, United States of America; 7 Department of Surgery, University of Chicago, Chicago, Illinois, United States of America; 8 Department of Radiation Oncology Duke University Medical Center, Durham, North Carolina, United States of America; Roswell Park Cancer Institute, United States of America

## Abstract

**Background:**

Cancer staging and treatment presumes a division into localized or metastatic disease. We proposed an intermediate state defined by ≤5 cumulative metastasis(es), termed oligometastases. In contrast to widespread polymetastases, oligometastatic patients may benefit from metastasis-directed local treatments. However, many patients who initially present with oligometastases progress to polymetastases. Predictors of progression could improve patient selection for metastasis-directed therapy.

**Methods:**

Here, we identified patterns of microRNA expression of tumor samples from oligometastatic patients treated with high-dose radiotherapy.

**Results:**

Patients who failed to develop polymetastases are characterized by unique prioritized features of a microRNA classifier that includes the microRNA-200 family. We created an oligometastatic-polymetastatic xenograft model in which the patient-derived microRNAs discriminated between the two metastatic outcomes. MicroRNA-200c enhancement in an oligometastatic cell line resulted in polymetastatic progression.

**Conclusions:**

These results demonstrate a biological basis for oligometastases and a potential for using microRNA expression to identify patients most likely to remain oligometastatic after metastasis-directed treatment.

## Introduction

Metastases are the leading cause of cancer death. Standard therapies for most metastatic cancers are systemic chemotherapy, hormonal manipulation or newer targeted therapies. However, these agents are rarely curative. We proposed that during the evolution of some tumors, an intermediate metastatic state exists called *oligometastasis(es)*. We hypothesized that these patients, exhibiting a less aggressive biology with limited [1], [2], [3] cumulative metastasis(es) in less than 4 months from time of first metastatic progression, could potentially benefit from metastasis-directed therapy [1], [3]. This hypothesis was based on long-term survival following surgical resection of limited lung [2], liver [4], [5], or adrenal metastases[6] from a variety of primary sites. An oligometastatic state is a common clinical presentation although it has only recently received attention as a defined subset of metastasis [1], [7], [8]. Employing radiotherapy improvements, termed hypofractionated image-guided radiotherapy (HIGRT) or stereotactic body radiotherapy (SBRT), we [9] and others [8] treated metastatic lesions using a few high-doses of radiotherapy in inoperable patients with ≤5 metastasis(es). Initial reports demonstrated long-term disease free survival in some treated patients [8], [9], [10], [11]. However, many oligometastatic patients developed widespread cancer progression and were subsequently classified as polymetastatic (>5 new metastatic sites, **see methods**). We hypothesized that molecular markers could be developed for identifying patients who would fail to become polymetastatic. We analyzed microRNA expression derived from paraffin blocks of patients who were oligometastatic at time of treatment with curative intent radiotherapy. We report unique prioritized features of a potential microRNA classifier associated with persistence in an oligometastatic state [1], [3]. We also confirmed that microRNA-200c, a top prioritized microRNA elevated in clinical polymetastases, regulates the conversion from oligo- to poly- metastasis(es) in an oligometastatic mouse model.

## Materials and Methods

### Patient population and clinical data

All human studies were carried out according to protocols approved by the Institutional Review Board (IRB) at the University of Chicago. Written consent forms were obtained from all participants involved in the study. Patients had 1–5 metastatic tumors that could be treated with hypofractionated radiation and encompassed in a conformal radiation field without undue expected toxicity based on size (<10 cm) or location. Patients underwent computed tomography based radiation treatment planning accounting for respiratory induced tumor motion and aided by intravenous and oral contrast media as needed. The attending radiation oncologist contoured tumors with no margin for microscopic extension using all available clinical, radiographic, and metabolic data then expanded 5–10 mm to account for set-up error. A variety of non-overlapping axial fields and non-coplanar fields were combined to achieve the optimal radiation distribution to tumors while minimizing radiation to surrounding non-involved organs. The estimated normal tissue tolerances from the available literature were referenced in determining radiation plans [8], [9], [12], [13]. Typically, radiation was delivered in three doses (8–16 Gy per dose) for those treated on protocol and in a ten-dose regimen (50 Gy total dose, 5 Gy per dose) for those treated off protocol. Furthermore, prospective level-1 evidence has demonstrated this approach, with or without whole brain radiotherapy (WBRT) [14], leads to 80–90% local control of lesions. From December 2004 to June 2010, 34 patients were treated with HIGRT at all sites of active limited metastatic disease [9] (**Tables S1, S2**). Eleven of these patients were analyzed retrospectively, while 23 patients were included prospectively from a previously reported radiotherapy protocol for oligometastasis(es) [9]. For inclusion in this report, availability of at least one formalin fixed paraffin embedded (FFPE) tissue biopsy from the primary site or a metastatic site was also required. Patients with small volume biopsies or fine needle aspirations were excluded, as there was not enough tissue for RNA extraction.

We collected paired primary and metastatic tumor samples from 5 patients, primary tumors only from 20 patients, and metastatic tumors only from 9 patients. Following radiotherapy, patients underwent physical examination and imaging (whole body CT and/or FDG/PET or MRI) at one month following HIGRT to assess initial response and then every three months subsequently for up to 41 months. Metastasis(es) were defined based on axial imaging using CT scans of the Chest/Abdomen/Pelvis with iodinated contrast. For brain imaging, gadolinium enhanced MRI scans was used. The modality chosen for follow-up was based on the imaging employed to initially evaluate and treat the patient”. The percentage of imaging modalities used to select and treat patients is included in **Table S5c**. Survival was defined as the time from the initiation of radiation treatment until death from any cause. Patients were classified into two groups based on response after completion of radiation therapy: *polymetastatic patients* had (i) progression in developing more than 5 new tumors in less than 4 months from time of first metastatic progression, or (ii) progression within a body cavity that by definition would imply the presence of diffuse metastatic disease (i.e. pericardial, pleural, cerebrospinal, or ascitic fluid). In contrast, *Oligometastatic (Oligo) patients* had either no evidence of progression (including 10 patients) or insufficient rate of metastatic progression to satisfy the above criteria for polymetastases.

### Human tissue acquisition, RNA extraction and microRNA profiling

After Institutional Review Board approval, FFPE primary and metastatic tissue samples were received in triplicate from the Department of Pathology at the University of Chicago. Total RNA was extracted from FFPE tissue samples using RecoverAll Total Nucleic Acid Isolation Kit (Applied Biosystems, Allston, MA, USA). Tissues of ≤80 µm were sectioned into sizes of 5–20 µm and underwent deparaffinization, protease digestion, nucleic acid isolation, and nuclease digestion/purification according to the manufacturer's protocol for RNA isolation. Sample concentrations were determined using the Qubit Quantification Platform (Invitrogen, Carlsbad, CA, USA) and normalized to 10 ng/µL.

Ten µL of each triplicate were combined and 3 µL of this pooled sample were used to obtain a total of 30 ng of total RNA. Single stranded cDNA synthesis and pre-amplification were performed according to the manufacturer's protocols (Applied Biosystems, Allston, MA, USA). Real-time qPCR of 376 distinct microRNAs was performed using human Taqman MicroRNA Array A Card v2.0 (Applied Biosystems, Allston, MA) according to the manufacturer's protocol.

### Differential microRNA expression for prioritization of oligo vs polymetastases from TaqMan Arrays

Among the 42 tumor samples included in the study, five patients had paired metastatic and primary tumor samples, while the remaining samples were from distinct patients with either primary or metastatic tumor tissue analyzed. In addition, 2 patients contributed samples from two distinct metastatic sites (**Tables S1, S2**). The raw Ct (threshold cycle) values and array qualities were analyzed and normalized using HTqPCR package in Bioconductor (**Methods S1**). Forty-two of the forty-five human samples assayed by TaqMan microRNA Card A for having more than 200 detectable microRNAs (Ct<38) were included in the analysis, while 3 samples with less than 120 detectable microRNAs were excluded (**Figure S3**). For the remaining 42 samples, quantile normalization was performed to control for potential genome-wide tissue/samples-specific bias. The coefficient of variation (CV) of external and endogenous controls was ≤5% after normalization. The raw Ct values normalized with the pooled controls of RNU-44 and RNU-48 were used to evaluate the impact of different normalization on our results. RNU-44 and RNU-88 are two small non-coding RNA (ncRNAs) that are expressed both abundantly and stably. They are widely used as endogenous control for microRNA expression profiling. Quantile normalization was applied to the datasets using default parameters of the R/Bioconductor package *HTqPCR* [15]. The raw and normalized TaqMan array data of these clinical samples have been deposited in the NCBI GEO database with accession number GSE25552.

Unsupervised hierarchical clustering was conducted using dChip software with the default parameters (“average” linkage and “1-Pearson” distance metric) [16]. The microRNA's expression profiles included in the unsupervised hierarchical clustering analyses had a standard deviation >0.5 across all samples regardless of the oligo- or polymetastastic status, resulting in the detection of 344, 335 and 330 out of 384 microRNA probes in primary only, metastatic only, and paired primary-metastatic datasets, respectively. This unbiased procedure removed uninformative microRNAs. The small sample sizes precluded achieving statistical significance after adjustment for multiple comparisons, thus deregulated microRNA expression of oligo- vs polymetastases groups in the metastatic samples and in the primary samples were “*prioritized*” using a two-tailed Student t-test at an unadjusted p-value <0.05 and organized according to their fold change. The prioritized microRNAs from primary sample, Pr-miRs, were used to predict the oligo- vs poly- metastatic progression in the metastatic samples using the default parameters and unsupervised Principal Component Analysis (PCA) of the R package “ade4” [17], [18]. Similarly, the prioritized microRNAs from the metastastatic tissue sample, M-miRs, were used to predict the oligo- vs poly- metastatic progression in the primary samples datasets. For permutation resampling of the samples, see **Methods S1**.

### Validation of prioritized oligo vs polymetastases microRNA signatures using independent datasets and ROC curves

MicroRNAs prioritized from primary tumors (Pr-miRs) and those prioritized from metastatic tumors (M-miRs) lists were used as features to compute the first component in these independent validation sets of microRNAs (Flow diagram of samples in **Figure S4**). **The clinical definitions of oligo- and polymetastatic progression are summarized in Figure S5.** PCA and 1^st^ component were calculated in the validation sets using the Pr-miRs and M-miRs microRNA lists. The computed first component was then used to generate an ROC (Receiver Operating Characteristic) curve using R “caTools” package of Bioconductor [16], [19] for the validation in human samples. The ROC curve plots the true positive rate against the false positive rate according to different possible thresholds for oligo vs polymetastases determination. An empirical p-value was calculated for the area under the curve (AUC) of the ROC by permutation resampling. In each permutation, class assignment of oligo or polymetastases was sampled without replacement in the validation sets. This simulation was performed 1000 times for metastatic tumor samples using Pr-miRs and likewise for primary tumor samples using M-miRs. We thus generated a conservative empirical distribution of AUCs for separating oligometastatic samples from polymetastatic samples using the 17 Pr-miRs and the 29 M-miRs, respectively. Scatter plots and non parametric Mann-Whitney tests were performed using GraphPad Prism version 4.03.

### Cell Cultures

Parental MDA-MB-435-GFP cell line was derived from the MDA-MB-435S (HTB-129) originally obtained from American Type Culture Collection (ATCC, Manassas, VA, USA). MDA-MB-435-GFP cell line authentication was performed by Fragment Analysis Facility, Johns Hopkins University (Baltimore, MD, USA) using Identifier AB Applied Biosystems. The STR profile perfectly matches that of MDA-MB-435S (HTB-129) in the ATCC database and there is no evidence of contamination with other cell types. MDA-MB-435-GFP cell line stably expressing green fluorescent protein (GFP) was generated by Dr. Robert Hoffman (AntiCancer Inc.) as previously described [20]. We have been using this model routinely to produce experimental lung metastasis(es) for conducting *in vivo* imaging experiments [21] (**data not shown**). Cells were maintained in DMEM high glucose supplemented with 10% FBS+200 µg/ml G418 (Gibco). B16F1 murine melanoma cell lines were obtained from ATCC (Manassas, VA, USA) and cultured in RPMI 1640 media (Invitrogen, Carlsbad, CA, USA) supplemented with 10% fetal bovine serum (Atlanta Biologicals, Lawrenceville, GA, USA). Cells were sub-cultured for at least three passages before harvesting at their linear growth phase (approximately 70–80% confluent) for tail vein tumor injection.

### Generation of derivative MDA-MB-435 lung oligometastatic (L1-R1) or polymetastatic (L1Mic-R1) cell lines from *in vivo* modeling of experimental lung colonization assays

All animal studies were carried out according to protocols approved by the IACUC Committee at the University of Chicago (Protocol ID#71685). The tail vein experimental lung colonization assay was performed to model the development of MDA-MB-435-GFP oligometastatic or polymetastatic phenotype in the lung and other organs *in vivo*. Animal work was conducted in accordance with an approved protocol. Age and weight-matched NCI athymic female mice were used, and 2×10^6^ viable cells were injected into the lateral tail vein. Animals were sacrificed once visible macroscopic metastatic lesions were identified upon external examination using Sellstrom Z87 fluorescence goggles and LDP 470 nm bright blue flashlight. Otherwise, metastatic colonization of recipient mouse lung and other organs by MDA-MB-435-GFP cells was determined and scored at 12 weeks post tumor cell injection, the experimental end-point.

To generate MDA-MB-435-GFP lung derivative cell lines that would produce oligo- and polymetastatic dissemination upon tail vein injection of the tumor cells, we first generated paired cell lines derived from the same lung tissue that were obtained from lung macrometastases (L1-R1) and from live tumor cells that resided in the macrometastasis-free component of the lung in the same animal (L1Mic-R1). Subsequently, we characterized the oligo- and polymetastatic potential of L1-R1 and L1Mic-R1 lung cell lines using the experimental lung metastasis assay (n = 15 per line). We then established derivative MDA-MB-435-GFP cell lines from distinct lungs of oligo- and poly-metastatic animals that received the injection of L1-R1 cells and L1Mic-R1-435-GFP cells, respectively. Tumor cells were purified via G418 antibiotic selection for GFP expression. All three distinct L1-R2- lung cell lines that we obtained and four of the six distinct L1Mic-R2-435-GFP cell lines were used for microRNA profiling (see below). We also conducted an additional round of lung experimental metastasis assays (n = 6 for each cell line, 4 cell lines for each phenotype) to confirm the stability of phenotypic separation of the three L1-R2 and four L1Mic-R2 cell lines we profiled.

### Quantitative RT-PCR analysis of microRNA expression of the L1-R2- and L1Mic-R2-435-GFP cell lines

Total RNA from three oligometastatic L1-R2- and four polymetastatic L1Mic-R2-435-GFP lung derivative cell lines was extracted and purified using TRIzol (Invitrogen, Carlsbad, CA, USA) according to manufacturer's instructions. Genome wide microRNA expression changes of 367 distinct mature human microRNAs between oligo and polymetastases cell lines was measured using TaqMan Human MicroRNA Array A card v2.0 (Applied Biosystems, Allston, MA, USA) according to the manufacturer's instructions. Raw data was imported and normalized using SDS RQ Manager software (Applied Biosystems, Allston, MA, USA). In our analysis, the baseline Ct was automatically set and we used a threshold of 0.3 for TaqMan raw data normalization. The raw and quantile normalized TaqMan array data of these clinical samples have been deposited in the NCBI GEO database with accession number GSE29890.

### *In vivo* assessment of the effect of microRNA-200c miRIDIAN mimics treatment on metastatic progression in two mouse models

40% confluent L1-R2-435-GFP cells or B16F1 cells were transfected with 100 nM Control mimics (Cat#110CN-001000-01), or species-specific miR-200c miRIDIAN mimics (L1-R2-435-GFP: #C-300646-05-0010; B16F1: # MIMAT0000039) (Dharmacon, Lafeyette, CO, USA) using Oligofectamine (Invitrogen, Carlsbad, CA, USA) as we previously described [22]. Transfection efficiency was optimized and estimated to be >90%. *In vivo* tail-vein injection of control or specific mimics-treated L1-R2-GFP (2×10^6^ cells/mouse) or B16F1 cells (1×10^5^ cells/mouse) was performed at 48 h after transfection.

For the L1-R2-435-GFP model, tumor-cell inoculated mice were monitored and scored for tumor metastasis development and progression as described above. For the B16F1 mouse melanoma model, 4–6 weeks C57BL/6 female mice were obtained from Harlan labs (Indianapolis, IN, USA). The care and treatment of experimental animals was in accordance with institutional guidelines at the University of Chicago. Mice were sacrificed 14 days after tail vein injections. The thoracic cavity of each mouse was opened and lungs were removed in their entirety and surface lung metastasis(es) were scored using methods previously described [23].

After being excised from each mouse, the lung tissue was fixed in 10% formalin, embedded in paraffin, cut into 5 micrometers sections, stained with hematoxylin and eosin and examined for macro- or micrometastases. 5 mice were examined from each group.

### TaqMan quantification of putative microRNA-200c gene targets expression

L1-R2-435-GFP cells were treated with equal amount of control-mimics or microRNA-200c mimics for 48 hours as described above. Thereafter, one fifth of the transfected cells were used for total RNA extraction and the rest were used for tail-vein injection. The expression of Zeb1 (Hs00232783_m1), Zeb2 (Hs00207691_m1), NEDD4 (Hs00406454_m1) and FGD1 (Hs00171676_m1) was determined by TaqMan RT-PCR assay according to manufacturer's instructions. GAPDH (4326317E) expression was used as normalization control.

## Results

To identify molecular changes associated with oligo or polymetastatic progression we extracted RNA from 42 paraffin embedded samples of primary and metastatic tumors of patients treated with stereotactic radiotherapy (see **Tables S1, S2** for patient characteristics) and profiled the resultant microRNAs using TaqMan Human MicroRNA Array A card v2.0 (see **Methods**). Among the 42 tumor samples included in the study, five patients had paired metastatic and primary tumor samples, while the remaining samples were from distinct patients with either primary or metastatic tumor tissue analyzed. In addition, 2 patients contributed samples from two distinct metastatic sites (**Tables S1, S2**). No differences were observed in pre-radiotherapy clinical variables (**Tables S3a–b**) or histopathology between patients who remained oligometastatic and those who progressed to a polymetastatic state (logit regression, data not shown). Median follow up time was significantly longer in patients who remained oligometastatic (**Tables S3, S4, S5**).

**Figure 1:**
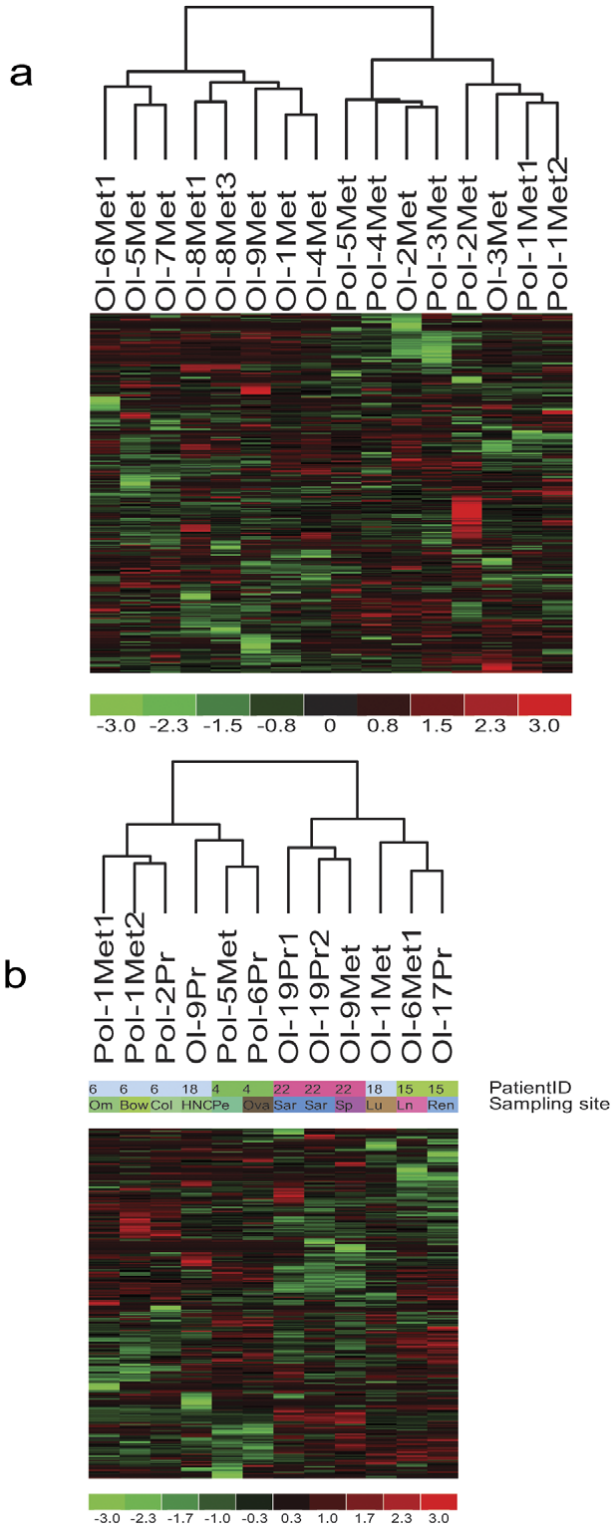
Unsupervised hierarchical clustering of: (**a**) metastatic tumors microRNA expression showing clustering of oligo- vs polymetastatic samples. Red, black and green represent TaqMan qPCR Ct values above, at or below mean level, respectively, across all samples and 335 microRNAs. As shown, all seven polymetastatic samples are clustered together, while eight out of ten oligometastatic samples cluster together. This suggests that the oligo vs polymetastatic phenotype is overriding other predictable groupings such as histology of primary tumor and metastatic site. However, in the primary samples, the primary site was the dominant signal of the unsupervised hierarchical clustering (**Fig. S1**). (**b**) MicroRNA expression of five patients with paired primary and metastatic samples showing clustering of (i) primary (Pr) and metastasis(es) sample sites of the same patient and (ii) oligo (Ol-) vs polymetastatic (Pol-) progression phenotype across patients.

Unsupervised hierarchical clustering of patients with metastatic tumor samples profiled correctly classified the clinical course of 8 of 10 (80%) samples from patients who remained oligometastatic and 6 of 6 (100%) samples from patients who eventually progressed to widespread, polymetastases (**Fig. 1a**, P = 0.007, two-tailed Fisher Exact Test). These data demonstrate that detected patterns of microRNA expression from metastatic samples are dominated by oligometastatic or polymetastatic progression of disease (**Fig. 1**, **Table S1**). In contrast, unsupervised hierarchical clustering using microRNA expression of tissue exclusively obtained from primary tumors of patients failed to accurately separate oligometastatic and polymetastatic patients (**Fig. S1**). Indeed, unsupervised methods are not designed to identify a phenotype, such as the subtle distinction between oligo- and poly-metastases, while the primary tumor cells are more heterogeneous than those of metastases. We thus obtained microRNA profiles of 5 patients for whom both primary and metastatic samples were collected. In four of five patients primary and metastatic tumor samples, the microRNA of the same patient clustered together consistent with other reports. Furthermore, in this paired sample analysis, the separation of oligometastatic *vs.* polymetastatic progression was confirmed both across different patients (**Fig. 1b**).

**Figure 2:**
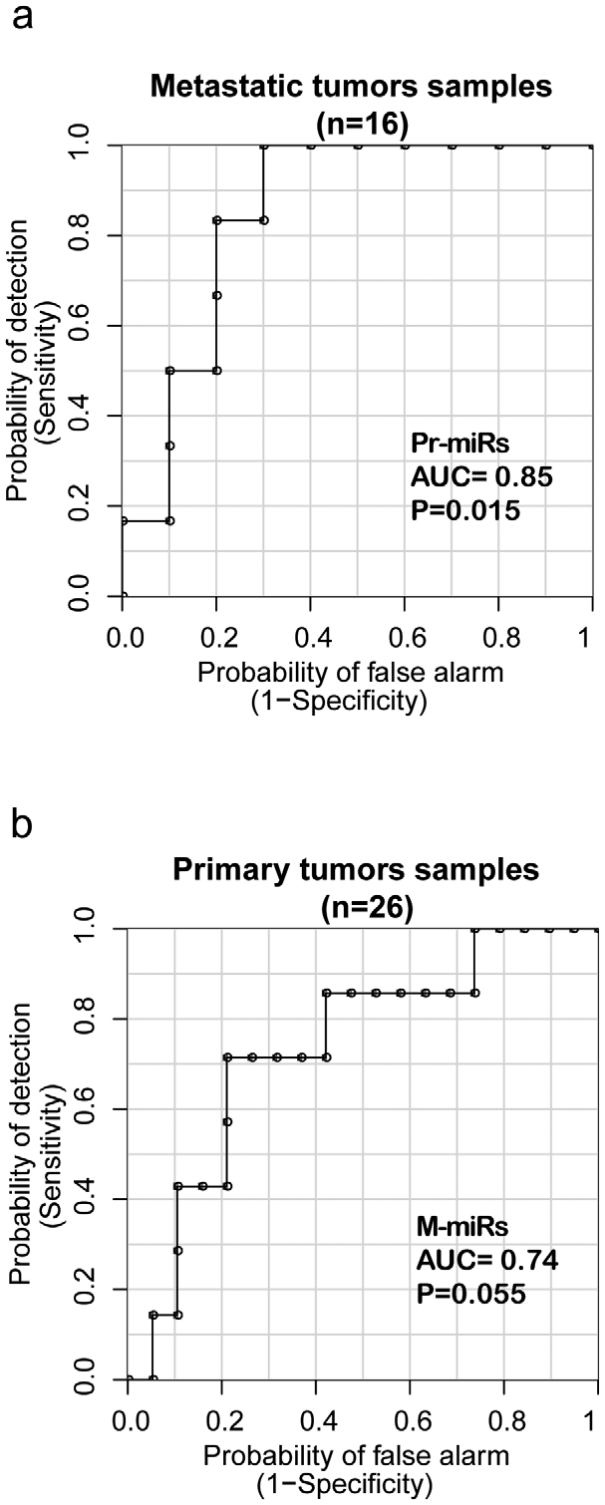
Validation of microRNA expression signatures in human datasets: prediction of oligometastatic progression by microRNA expression signatures. The Receiver Operating Characteristic (ROC) curves describe how accurately the prioritized microRNAs can discriminate between oligo- vs poly- metastasis(es) samples by plotting the possible combinations of sensitivity and specificity obtained at different cutoff points of the prioritized microRNA classifier. (**a**) Pr-miRs, 17 prioritized microRNAs from the primary tumors sample (**Table 1b**), were used to predict oligometastasis(es) progression in the 16 metastatic tumor samples using permutation controlled ROC curves of the first PCA component (See **Methods**). (**b**) Similarly, M-miRs, 29 prioritized microRNAs from the metastatic tumor samples (**Table 1a**), were used to predict oligometastasis(es) progression in the 26 primary samples. Empirical P values of the AUC were calculated from empirical permutation resampling (see **Methods S1**).

To derive microRNA expression patterns associated with patients remaining oligometastatic versus progressing to polymetastases, we compared expression of individual microRNAs between the oligometastatic and polymetastatic groups in the metastatic tumor dataset and the primary tumor set independently using a two-tailed Student t-test (P<0.05). We prioritized 29 and 17 microRNAs that characterized oligometastatic or polymetastatic progression in the two datasets, respectively (**Table 1** **,** **Fig. 1** **, Figure S1**). We designated these sets as 29 M-miRs (microRNAs prioritized from metastatic tumors, **Table 1a**) and 17 Pr-miRs (microRNAs prioritized from primary tumors, **Table 1b**). To validate Pr-miR and M-miR, we applied them to patients in the alternative dataset (ie Pr-miR was tested in the patients with metastatic tissue obtained and M-miR in the patients with primary tissue profiled). This analysis was performed using the unsupervised first component of a principal component analysis (PCA) (**Methods, Methods S1**). At different cutoff points of the unbiased Pr-miRs and M-miR-derived classifiers, the combinations of sensitivities and specificities reflect their ability to discriminate between the oligo- vs polymetastatic tissue samples thus are plotted as receiver operating characteristic (ROC) curves in **Fig. 2**. The resulting prioritized microRNAs from primary samples, Pr-miRs, demonstrate good discrimination between remaining oligometastatic and developing widespread polymetastases in the metastatic sample set (**Fig. 2a**, AUC = 0.85; empirical P = 0.015 by permutation resampling). Similarly, M-miRs applied to the group of primary tumors discriminated between the two phenotypes in primary tumors (**Fig. 2b**; AUC = 0.74, empirical P = 0.055).

**Table 1:** a and b. Prioritized microRNAs by Expression Analysis of Oligo- vs Polymetastases in Human Metastatic and Primary Tumors.

Table 1a. Oligo vs polymetastases progression in metastatic tumor samples (M-miRs)					
MicroRNA	FC	p (t-test)	MicroRNA	FC	p (t-test)
miR-654-3p	28.3	0.028	miR-95	2.4	0.029
miR-654-5p	24.6	0.041	miR-500	−2.1	0.047
miR-200c	20.1	0.029	miR-328	−2.2	0.002
miR-105	15.9	0.023	miR-125a-3p	−2.2	0.048
miR-375	14.9	0.027	miR-140-5p	−2.2	0.024
miR-135b	7.8	0.013	miR-29c	−2.4	0.008
miR-200b	5.7	0.032	miR-140-3p	−2.4	0.018
miR-410	5.4	0.01	miR-489	−2.7	0.008
miR-376a	4.7	0.049	miR-331-5p	−3.6	0.046
miR-323-3p	4.1	0.023	miR-193a-3p	−6.7	0.036
miR-539	4	0.045	miR-199b-5p	−9.5	0.043
miR-642	3.6	0.024	miR-502-5p	−18.3	0.034
miR-370	3.2	0.031	miR-545	−20.2	0.022
miR-127-3p	3	0.04	miR-363	−21.6	0.012
miR-212	2.7	0.002			

Table 1b. Oligo vs polymetastases progression in primary tumor samples (Pr-miRs)					
MicroRNA	FC	p (t-test)	MicroRNA	FC	p (t-test)
miR-654-3p	17	0.018	miR-127-3p	1.7	0.036
miR-542-3p	14.3	0.014	miR-24	−1.5	0.014
miR-548c-3p	10	0.001	miR-27b	−1.6	0.025
miR-758	8.8	0.045	miR-197	−1.9	0.032
miR-483-5p	3.6	0.038	miR-330-3p	−2	0.01
miR-369-3p	3.6	0.047	miR-671-3p	−2.2	0.012
miR-134	2.5	0.023	miR-23b	−2.8	0.048
miR-337-5p	2.3	0.027	miR-301b	−16.8	0.007
miR-181a	2	0.034			

Prioritized microRNAs by comparing their expression in oligo- and polymetastatic groups using Student t-test (unadjusted p<5%). A positive fold change (FC) represent elevated expression in polymetastatic progression as compared to oligometastasis(es).

**Figure 3:**
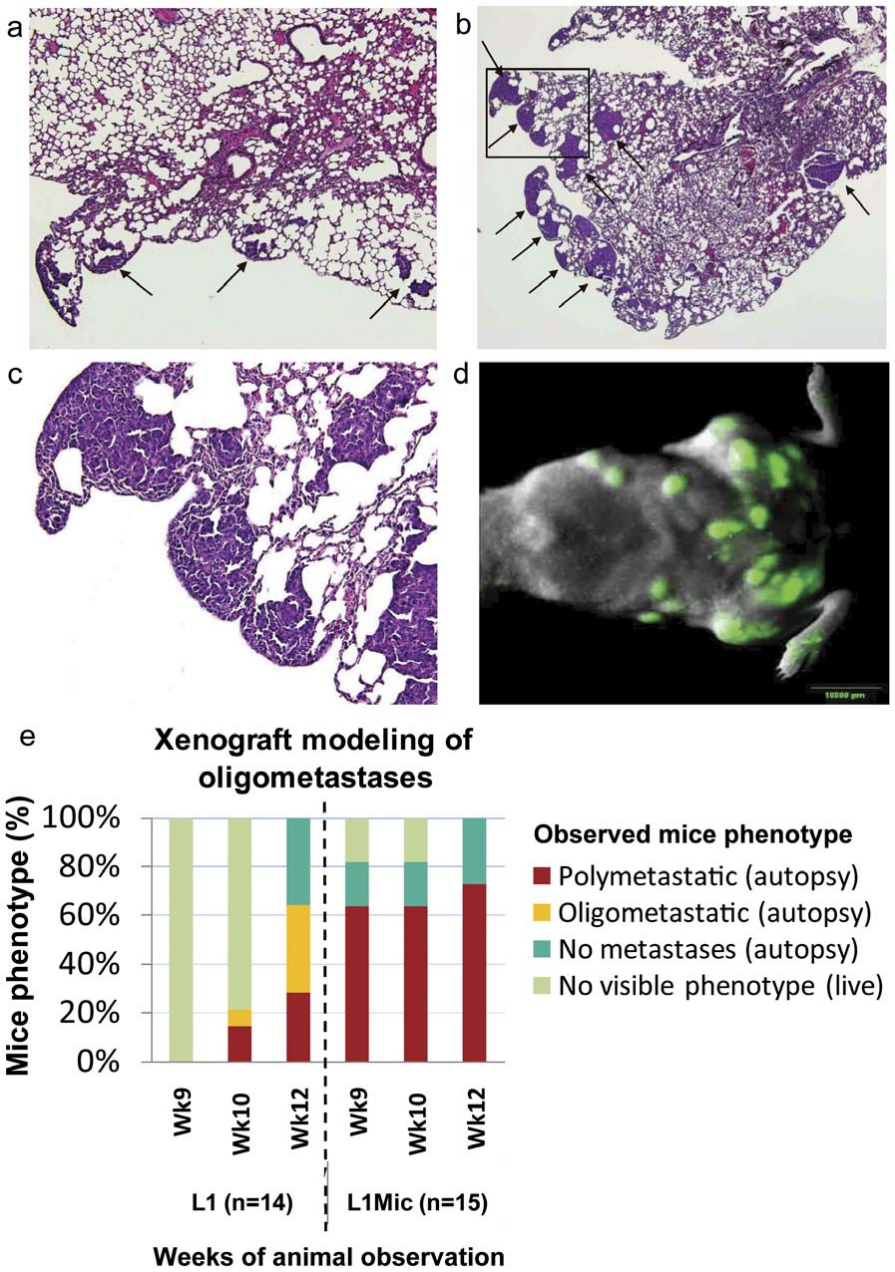
Histological and *in vivo* characterization of oligo- and poly- metastasis(es) derived from tail-vein injected MDA-MB-435-GFP lung derivative cell lines. 2×10^6^ purified MDA-MB-435-GFP lung derivative cell lines established from lungs harboring oligo- (L1-R1) or poly-(L1Mic-R1) metastases respectively were injected via tail-vein. Animals developing macroscopic observable metastases were sacrificed at the time of this finding. The rest of the animals were sacrificed at 12-weeks post tumor cell injection. Necropsy was performed to score macroscopic metastatic lesions and lungs were harvested and paraffin embedded for histological characterization. (**a**) Representative lung metastatic-foci developed from oligmetastatic L1-R1 cell line harvested at week-12 or (**b**) a polymetastatic L1Mic-R1 cell line, harvested at week-7 shown by H&E staining (arrows, 40× magnification). (**c**) An enlargement (200×) of the insert in (**b**). (**d**) Representative fluorescent *in vivo* imaging identifying extensive lung and whole body polymetastatic lesions after tail vein injection with L1Mic-R1 cells (OV-100 imager, green fluorescence = metastatic lesions). (**e**) Oligo- vs polymetastases progression in these 29 NCI athymic female mice establish that polymetastatic L1Mic-R1 cells produced more aggressive metastatic progression than the oligometastatic L1-R1 cells (odds ratio at week 12 = 10; P = 0.0092; two-tailed Fischer Exact Test). Additionally, L1Mic-R1 produced more aggressive metastatic progression: at week 9, 73% of L1Mic-R1 had developed polymetastases as compared to none among those exposed to L1-R1 (P = 5×10^−5^; two-tailed Fischer Exact Test).

**Figure 4:**
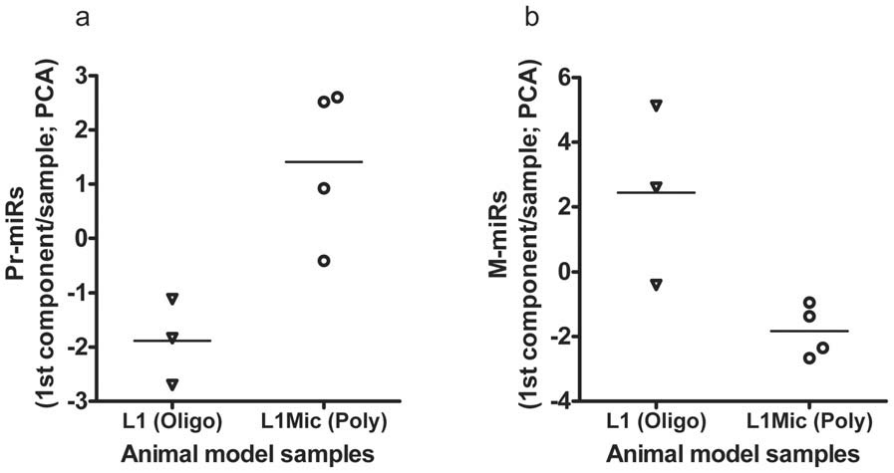
Validations of the prioritized human microRNAs in the animal model of oligo and polymetastases. The prioritized microRNAs between oligometastatic and polymetastatic progression were identified in primary tumors and in metastatic tumors of clinical samples yielding two lists: Pr-miRs and M-miRs, respectively (see **Table 1a–b**). These lists of microRNAs were used to rank the microRNA expression of seven cell line samples derived from animal modeling of oligometastasis(es) (L1-R1) and of widespread polymetastases (L1Mic-R1). MicroRNA expression was conducted in three oligometastatic L1-R2 lung cell lines as well as four polymetastatic L1Mic-R2 lung cell lines from seven distinct animals. Principal component analysis of the expression of microRNAs was conducted in these cell line samples without providing any information on the L1-R2 or L1Mic-R2 status. In each sample, the first component values of (**a**) Pr-miRs and of (**b**) M-miRs is sufficient to discriminate between the oligo- (L1) and polymetastatic (L1Mic) phenotype of the animal model (Pr-miRs P = 0.058; M-miRs P = 0.058; two-tailed Mann-Whitney U Test, **Methods S1**).

Since differentially expressed microRNA profiles were generated from a relatively small patient cohort, we developed a stable human tumor (MDA-MB-435-GFP) xenograft model of oligometastatic and polymetastatic progression by conducting three consecutive rounds of experimental lung colonization assays (see **Methods**). In the first round, we generated paired oligometastases-like lung derivative L1-R1-435-GFP (L1-R1) or polymetastases-like L1Mic-R1-435-GFP (L1Mic-R1) cell lines. When tested *in vivo*, these cells stably recapitulated human oligometastatic (≤5 total metastasis(es) in mouse) and polymetastatic (>5 metastases in mouse) states at week 12 in subsequent testing (**Fig. 3a–e**, **Fig. S2**, see **Methods**). For example, in the second round (fifteen mice for each cell line), L1Mic-R1 cells produced widespread polymetastases in the lung and other organs at a higher incidence and had significantly faster time kinetics of metastatic dissemination than the oligo-like L1-R1 cell line (odds ratio of poly = 10 at week 12: P = 0.0092, two-tailed Fisher's exact test; time kinetics at week 9: P = 5×10^−5^, two-tailed FET; **Fig. 3e**). We subsequently generated three oligometastatic L1-R2-435-GFP (L1-R2) lung cell lines as well as four polymetastatic L1Mic-R2-435-GFP (L1Mic-R2) lung cell lines from seven distinct animals of the second *in vivo* passage for further biological characterization and for microRNA expression analysis (see **Methods**, **Fig. 3e**, **Fig. S2**). PCA using the first component shows that the prioritized Pr-miRs and M-miRs (**Table 1a–b**) accurately split the MDA-MB-435 lung derivative cell lines into oligometastatic L1-R2 and polymetastatic L1Mic-R2 groups. These observations have provided further evidence that distinct microRNA expression patterns derived from patients underlie the molecular differences between the stable oligometastatic phenotype and that of polymetastatic progression (**Fig. 4a–b**).

Next, we investigated whether specific microRNAs differentially expressed between oligometastatic and polymetastatic patients were associated with phenotypic change from oligo- to polymetastases. Since metastatic development is a multi-step process and all patients by definition had 1–5 metastasis(es) at time of radiation treatment, we hypothesized that late events in the metastatic process were likely to account for differences in the oligo- and polymetastastic phenotypes. Primary tumors are likely more heterogeneous with respect to cells with metastatic potential [24], thus we focused on the prioritized microRNAs derived from the metastatic tissue samples. We rank ordered the 29 prioritized microRNAs obtained from metastatic tissue according to fold change. As shown in **Table 1b**, the two microRNAs with highest fold changes, miR-654-3p and miR-654-5p, are produced in the cells by two-complementary/opposite strands of the same precursor microRNAs. Their joint expression suggests a common transcriptional event likely unrelated to their specific function. These microRNAs are also not well characterized. We therefore investigated the microRNA with the next highest fold change, microRNA-200c (**Table 1a**, FC = 20.1, p = 0.029), as proof of principle that these microRNAs mediate the oligo- to polymetastatic progression. MicroRNA-200c, along with other members of the microRNA-200 family including microRNA-200b, (**Table 1a**, FC = 5.7, P = 0.032) has been widely reported to be involved in metastasis [25], [26], [27]. MicroRNA-200c has anti- or pro-metastatic functions depending on at which point in the metastatic cascade it acts. For example, it inhibits the invasiveness of cancer cells at the primary site by suppressing epithelial to mesenchymal transition (EMT) [28], while it enhances colonization efficiency at distant metastatic sites by promoting the reversion from EMT to mesenchymal-to-epithelial-transition [27], [29].

**Figure 5:**
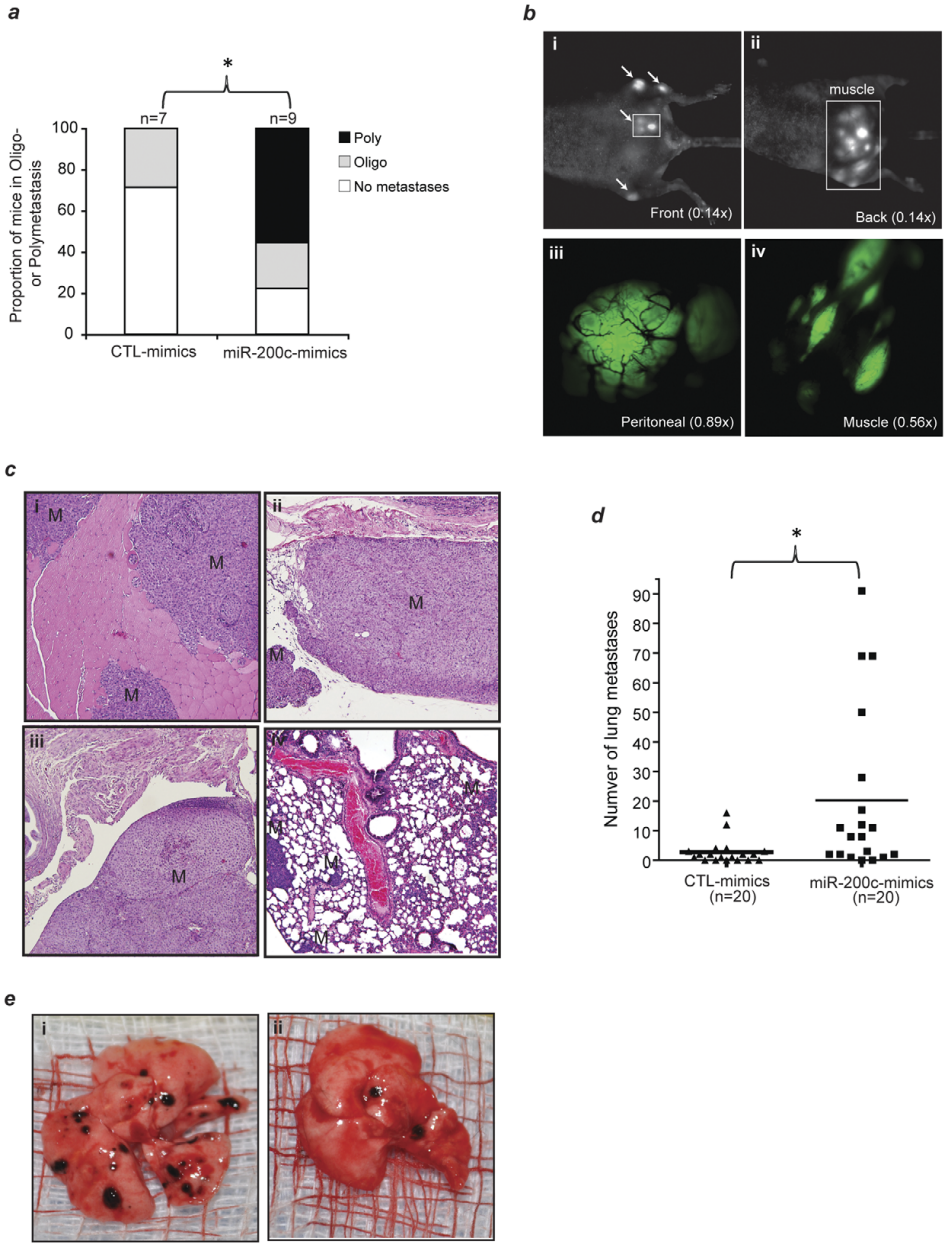
microRNA-200c regulate oligo- to poly- metastasis(es) progression in the L1-R2-435-GFP xenograft model. 2×10^6^ control-mimics or microRNA-200c specific mimics-treated L1-R2-435-GFP cells were tail-vein injected after 48 hr of transfection, and the development of macrometastases was monitored (Methods). (**a**) microRNA-200c mimics treatment significantly converted oligometastasis(es) to largely polymetastases. Poly: polymetastases; Oligo: oligometastasis(es). *P = 0.012 (one-tailed Mann Whitney U Test). (**b**) Non-invasive, variable magnification (0.14–0.89×) OV-100 fluorescent imaging visualization of polymetastatic dissemination in a representative animal injected with microRNA-200c mimics-treated L1-R2 cells. Arrows: macrometastases; green: L1-R2-435-GFP tumor; black lines in (iii): tumor blood vessels. (**c**) IHC confirmation of macrometastases in the muscle (i), peritoneum membrane (ii), peritoneal cavity (iii) and lung (iv). Magnification: 100×; M: macrometastases. (**d**) microRNA-200c mimics treatment significantly increased the efficiency of B16F1 mouse melanoma cells to form lung macrometastases. *P = 0.0057 (one-tailed Mann Whitney U Test). (**e**) Representative images of mouse lung obtained from animals tail vein-injected with microRNA-200c mimics treated (i) and control mimics treated (ii) B16F1 cells.

To demonstrate prioritized microRNAs from the clinical samples are functionally important, as a proof of principle, we examined whether microRNA-200c may regulate oligo- to polymetastatic progression. We specifically enhanced the function of this microRNA via synthetic mimics (**see Methods**) in the most stable oligo-like L1-R2 cell line prior to tail vein injection. Whereas injection of non-treated or control mimics-treated L1-R2 cells produced predominantly oligometastases or no macroscopic metastasis(es) (**Fig. 5a**, Oligo: non-treated = 2, control mimics = 2; no metastasis(es): non-treated = 3, control mimics = 5; poly = 0), increased expression of microRNA-200c in the L1-R2 cell line produced significantly more mice with polymetastases (**Fig. 5a**, oligo = 2; no metastasis(es) = 2; poly = 5; P = 0.012, one-tailed Mann Whitney U, for polymetastases compared to controls). Real-time imaging visualization and histological characterization also confirmed this conversion (**Fig. 5b–c**).

Since microRNA-200c has mainly been characterized as a metastasis suppressor, our prediction of its role in promoting oligo- to polymetastatic progression is novel. To further examine the pro-metastasis role of microRNA-200c, we also enhanced its function in the melanoma cell line B16F1 that has low metastatic propensity. Similar to our observations in the L1-R2-435-GFP xenograft model, treatment of B16F1 cells with microRNA-200c mimics resulted in significantly more macroscopic lung metastases than the control mimics-treated cells in a syngeneic mouse model. The average number of surface lung metastases per mouse was 2.8 versus 20.3 at 2 weeks (P = 0.0057, one-tailed Mann Whitney U Test) for controls and microRNA-200c mimics respectively (**Fig. 5d–e**). These results demonstrate significant increases in lung colonization efficiency due to enhancement of microRNA-200c function (**Fig. 5d**).

**Figure 6:**
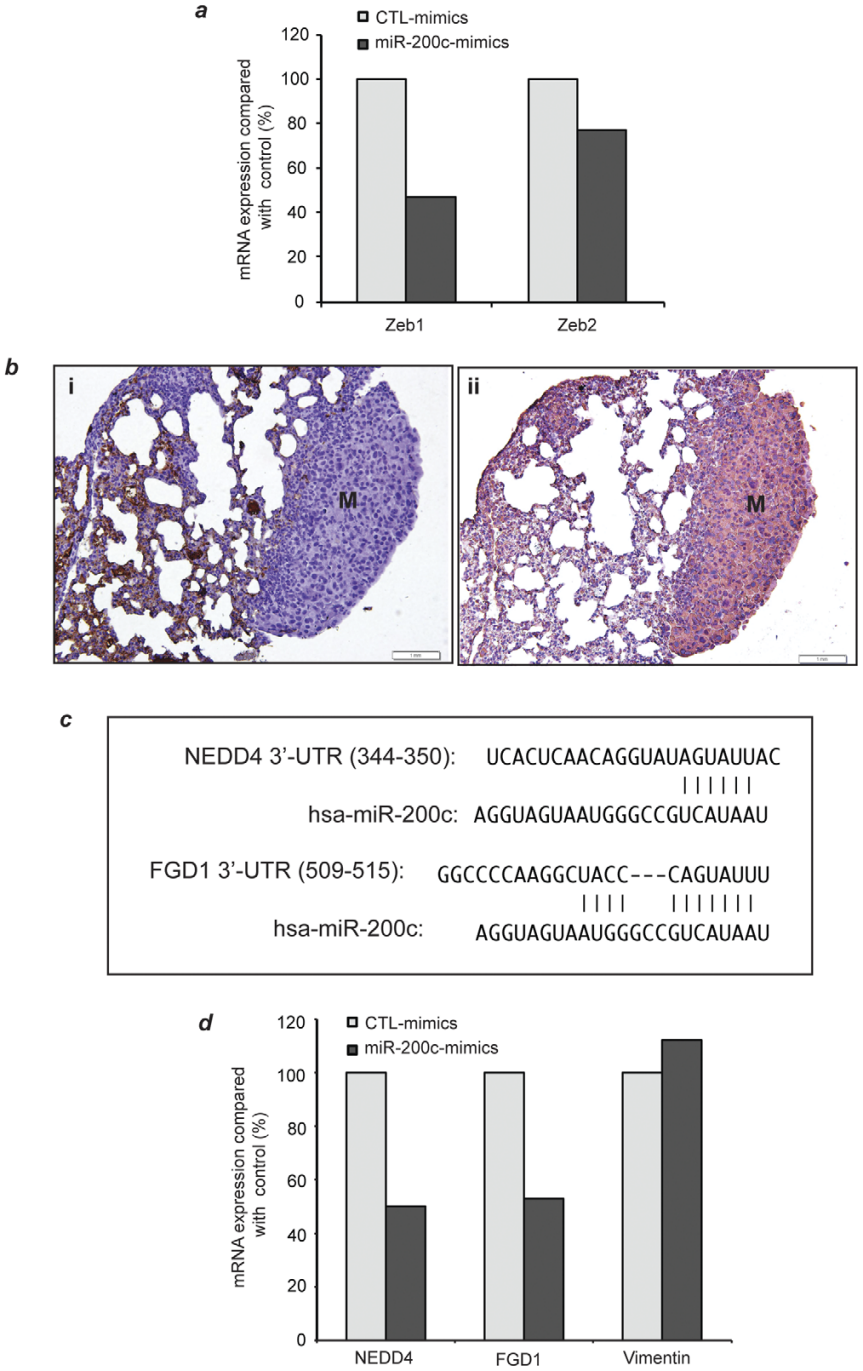
microRNA-200c mimics treatment lead to specific inhibition of its putative target gene expression. L1-R2-435-GFP cells were treated with equal amount of control-mimics or microRNA-200c mimics for 48 hours (Method). Thereafter, one fifth of the transfected cells were used for total RNA extraction and the rest were used for tail-vein injection (**Figure 5**). (**a**) TaqMan quantification of Zeb1 and Zeb2 mRNA expression. GPDH was used for normalization. (**b**) Lungs macrometastases derived from L1-R2-435-GFP cells treated with control mimics or microRNA-200c mimics were negative for E-cadherin (i) and positive for the EMT marker vimentin (ii). (**c**) TargetScan alignment of microRNA-200c binding site at 3′-UTR of two computationally prioritized microRNA-200c putative targets NEDD4 and FGD1. (**d**) TaqMan quantification of NEDD4, FGD1 and Vimentin mRNA expression. GPDH was used for normalization.

To determine the specificity of microRNA-200c in mediating the observed phenotype switch, we examined messenger RNA (mRNA) expression of Zeb1 and Zeb2 by Taqman RT-PCR in tail vein injected L2-R2 cells that were treated with microRNA-200c mimics. These two genes are validated microRNA 200c targets [26], [30]. In microRNA-200c mimics treated L1-R2 cells, the expression Zeb1 and Zeb2 was decreased by 53% and 23%, respectively compared to the control mimics-treated cells (**Fig. 6a**) confirming target specificity. Since one mechanism by which ZEB promotes EMT state is through transcriptional suppression of E-cadherin expression [25], [26], [31], and L1-R2-435-GFP cell lines were negative for E-cadherin [**Fig. 6b(i)**] and positive for vimentin [**Fig. 6b(ii)**], we searched for additional putative microRNA-200c gene targets that are validated regulators of EMT or metastasis.

We computationally prioritized putative, functional microRNA-200c gene targets in the L1- and L1Mic-435-GFP models by combining the 681 sequence alignment predicted targets of microRNA-200c from TargetScan with microRNA and gene expression analysis of putative gene targets expressed in the lung derivative oligo- or polymetastatic cell lines (L1-R2 vs L1Mic-R2) as well as xenograft lung metastases (L1-R3 vs L1Mic-R3) (see **Methods S1**). Of the 681 putative targets from TargetScan, 180 showed anti-correlation with microRNA-200c expression. Only three of these genes were significantly and differentially expressed between oligo and polymetastatic cell lines: FGD1 and USP25 from xenograft lung metastases and NEDD4L from lung cell lines. We chose NEDD4 and FGD1 for validation of microRNA-200c targeting based on their reported role in regulating EMT via TGF-ß signaling and Rho signaling, respectively [32], [33]. Shown in **Fig. 6c**, NEDD4 and FGD1 each contain a putative binding site for the microRNA-200 family members including microRNA-200c. As expected, the expression of these two genes in microRNA-200c mimics-treated L1-R2 cells was inhibited by 47% and 50%, respectively compared with that in control-mimics treated cells (**Fig. 6d**). In contrast, the expression of vimentin, a non-putative microRNA gene target, was not significantly altered (**Fig. 6d**). These findings further strengthen the targeting specificity of microRNA-200c and identify potential alternative EMT-regulatory pathways in cancer cells that have lost E-cadherin expression due to epigenetic modifications such as the DNA methylation.

We determined the expression of the epithelial marker E-cadherin (CDH1) and the mesenchymal marker vimentin in 5 polymetastatic and 8 oligometastatic samples (**Table S1**) by immunohistochemistry. Consistent with the elevated miR-200c expression in polymetastases and the anticipated inhibition of EMT (**Table 1**), vimentin expression was detectable in 6 of the 8 oligometastatic samples and in none of the polymetastases (P = 0.016, Fischer Exact Test). CDH1 was expressed in all polymetastatic tissue samples and in 6 of the oligometastatic tissue samples.

## Discussion

We have previously proposed oligometastases as a potentially curable state existing between absent and widespread metastases [1], [3]. While clinical outcomes data support the existence of oligometastasis [2], [4], [5], [6], [9], our prioritized microRNA expression is an initial step in demonstrating a molecular basis for this phenotype and may allow discrimination between patients with persistent oligometastasis(es) and those who will manifest polymetastatic progression. Our results differ from previous studies of differential microRNA expression between non-metastatic and widely metastatic states, or between primary and metastatic tissue within the same subject, because our results identify characteristics of an intermediate metastatic phenotype [34], [35], [36], [37], [38], [39], [40], [41], [42]. The oligometastatic versus polymetastatic phenotype emerges from metastatic tissue samples as the dominant unsupervised pattern of microRNA expression following unsupervised analysis of all microRNAs. This pattern derives from diverse primary histologies and metastatic sites suggesting a common molecular basis for maintaining an oligometastatic state across a broad variety of solid tumors. This pattern is not found in unsupervised analysis of primary tumors likely due to the increased genetic heterogeneity of the primary tumor samples compared to the clonal selection present in metastatic sites [24], [35], [43], [44], [45], though heterogeneity of cells have also been observed over their progression at their metastatic site [46]. Further, prioritized microRNAs from differential expression between oligo- and poly- metastasis(es) progression in primary samples predicted these phenotype in metastasis(es) samples (p = 0.015) of independent patients, while microRNAs prioritized from the metastases were less predictive in primary samples (p = 0.055) possibly due to the heterogeneity of the latter. A limitation of our study is a relatively small human tissue sample size. However we succeeded in developing prioritized features of a microRNA classifier of oligometastasis(es) for future clinical validation and testing. Additionally, the internal consistency between several different methods of analysis, the discrimination of oligo- and polymetastases in the L1/-L1Mic-435-GFP animal model, as well as the ability of microRNA-200c to convert stable oligometastasis(es) to polymetastatic progression in L1-R2-435-GFP xenograft model, and to enhance the lung colonization efficiency of B16F1 syngeneic model strengthen the validity of our clinical findings. While more investigation is necessary to identify the roles and gene targets that separate oligometastasis(es) from widespread disease, our data provide evidence for the molecular basis of oligometastasis(es) and represent a first step of investigation in what is likely to be a highly complex phenotype. Although these tumors represent different histologic subtypes, they bear similarities in biological behavior. The overlapping patterns of microRNAs that we have prioritized are consistent with the fact that common biological properties (e.g. invasion, metastasis) are shared by histologically heterogeneous tumors during disease progression.

These results are of clinical significance because limited metastatic disease is more common than generally recognized [7]. For example, potentially 50% of patients with metastatic non-small cell lung cancer, the leading cause of cancer death in men and women, may be oligometastatic [7]. However, despite using clinical characteristics to optimize patient selection for surgical/radiotherapeutic intervention, only approximately 25% of oligometastatic patients will experience long-term disease control with aggressive treatment of limited metastatic disease [2], [4], [5]. Identification of this subset may be enhanced by using molecular selection criteria, which could enrich the therapeutic benefit of metastasis-directed therapy, while redirecting patients unlikely to benefit from surgery or radiotherapy to systemic treatments. Similarly, patients with metastatic disease that at first presentation would appear not amenable to local treatment but exhibit an oligometastatic genotype might benefit from a combined aggressive local and systemic approach.

The direction of the prioritized microR-200c expression changes in our clinical data sets differs from reports analyzing expression patterns in non-metastatic versus metastatic patients. For example, 2 of the 5 microRNAs in the microRNA-200 family (miR-200b and miR-200c) are expressed at significantly higher levels in metastatic tissues from oligometastatic patients who progress to polymetastases compared to those who remain with oligometastasis(es) (**Table 1a**). Our investigation of the role of microRNA-200c in regulating oligometastatic to polymetastatic progression in the L1-R2-435-GFP xenograft model, as well as in regulating colonization efficiency in the B16F1 syngeneic model has provided new biological evidence for the emerging pro-metastasis role of microRNA200c [27] that was initially shown to suppress metastatic dissemination [47]. The availability of our clinically relevant animal models of oligo- and polymetastases has advantages over other current experimental metastatic models that were not designed to maintain a stable oligometastatic state during consecutive rounds of *in vivo* selection.

Our knowledge about the role of the miR-200 family continues to evolve. Many investigators have established that in tumorigenesis, one of the fundamental roles of the miR-200 family is to maintain an epithelial phenotype (i.e., preventing epithelial-to-mesenchymal transition) via its gene targets Zeb1 and Zeb2, the transcriptional suppressors of E-cadherin [25], [26], thus preventing a cancer cell from initiating the process of metastasis. When examined in the role of preventing cancer progression, investigators have shown that expression of this microRNA family can prevent a primary tumor from initiating metastasis by maintaining an epithelial phenotype [48]. However more recently there have also been studies suggesting that expression of the miR-200 family is associated with efficient metastatic colonization [27], [49], [50]. In their isogenic mouse model Dykxhoorn and colleagues have shown that after cancer cells acquire the ability to metastasize, they cannot efficiently form metastatic lung colonies without the expression of the miR-200 family [27]. Furthermore Elson-Schwab et al have shown that expression of miR-200c confers a cellular morphology that favors invasion and metastasis [50]. Finally Korpal and colleagues recently reported that miR-200s play a critical role in promoting the latter steps of metastatic colonization by targeting secretomes involved in metastasis suppression. In line with these studies, our study examines the miR-200 family in the context of a cancer cell after it has acquired the ability to metastasize. Our study is the first to report that expression of miR-200c is important in the segregation of the oligometastatic and polymetastatic states. Taken together, our study and those of others show that phenotypes representative of miR-200c expression vary in relation to the cellular context to which they are examined.

Another novel set of observations derived from our xenograft validation of microRNA-200c function is that we have identified two new putative gene targets of microRNA-200c that may also mediate regulation of EMT, in addition to the characterized Zeb 1/Zeb2/E-cadherin pathway. Nedd4 has been shown to inhibit TGF-ß signal by degrading TGF-ß activated Smads and/or TGF-ß Type 1 receptors [32], [51]. Thus, down-regulation of NEDD4 by microRNA-200c (**Fig. 6e**) will release its inhibition on TGF-ß cascade allowing TGF-ß to function as a metastasis promoter [52], [53], [54]. Therefore the interaction between Nedd4/TGF-ß pathway and microRNA-200c network may represent an alternative mechanism underlying the plasticity of an EMT state during metastasis [55], [56]. These data highlight the complexity of microRNAs in the control of the metastatic phenotype and represent new opportunities for future investigations.

In summary, we have identified microRNA expression features of a potential classifier that predict the distinct outcomes of metastatic patients who maintained stable oligometastatic disease from those who progressed to polymetastases. We also provide biological confirmation for molecular differences, in this case the microRNA regulation, that underlie oligometastic to polymetastatic progression.

## Supporting Information

Figure S1 **Unsupervised hierarchical clustering of primary tumors using the 344 microRNAs filtered from TaqMan miRNA card-A (Methods).** Red, black and green represent threshold cycle values above, at or below mean level across all samples. As expected, primary samples were clustered according to the tissue origin and sampling site rather than their oligo or polymetastases classifier. Abbreviations for sampling site: Col = Colon; HNC = Head and Neck carcinoma; Ren = Renal; Lu = Lung; Bre = Breast; Bla = Bladder; Sar = Sarcoma; Liv = Liver; Rec = Rectum; Bow = Small bowel; Che = Chest; Ova = Ovarian; Par = Parotid; Thy = Thymus.
(PDF)

Figure S2 **Verification of the phenotypic stability of the seven arrayed 2^nd^ generation cell lines via 3rd round of animal modeling.** 2×10^6^ purified lung derivative cell lines established from lungs of mice described in Figure 3 and for which the expression was determined (**Fig. 4**), were injected via tail-vein of 39 NCI female athymic mice (3 oligometastatic L1 and 4 polymetastatic L1Mic cell lines). Animals developing macroscopic observable metastases were sacrificed at the time of this finding. The rest of the animals were sacrificed at 12-weeks post tumor cell injection. Necropsy was performed to score macroscopic metastatic lesions and lungs were harvested and paraffin embedded for histological characterization. While the histology and clinical data reported in **Figure 3** refers to the cell lines extracted from lungs at generation two and arrayed, the data reported in this **Figure S3** pertain to animals injected with this second generation of cell lines (third round of animal modeling). In mice, the polymetastases MDA-MB-435-GFP-L1Mic cells lines produced more aggressive metastatic progression than the oligometastases MDA-MB-435-GFP-L1 ones in this third animal passage (odds ratio at week 12 = 5.6; P = 0.015; one-tailed FET).
(PDF)

Figure S3 **Quality of microRNA measurement in each human samples.** As a control of microRNA quality measure, the number of detectable microRNAs per sample was plotted using the Bioconductor package HTqPCR. Array ID 5a, 15c, and 49b are excluded from the current study because of their excessive number of undetectable microRNAs. Further experiment by PCR of two genes validated the RNA.
(PDF)

Figure S4 **The sources of individual samples, each representing a separate lesion is shown.** The * represents a single sample excluded because of excessive undetected microRNAs.
(PDF)

Figure S5 **Definitions of oligo- and poly- metastatic progression.**
(PDF)

Table S1 **Description of patient characteristics for the metastatic samples ordered by patient ID.** Number of metastasis(es) are listed as cumulative numbers since discovery of primary at the time of “radiation” or of “tissue sampling”. Time to metastasis(es) is defined as time to development of metastasis(es) after primary cancer diagnosis. Regional nodal metastasis(es) are not included in this study and all nodal sites listed represent distant metastases(‡). Metastasis(es) needed to be visible on CT or MRI at the time of radiotherapy. The total number of metastasis(es) was limited to ≤5 at the onset of the initial evaluation for treatment. During the follow-up period, patients who remained classified with the oligometastatic state demonstrated a cumulative number of metastasis(es) from 1 to 5 and did not have pericardial, pleural, cerebrospinal, or ascitic fluid. All reported count of metastasis(es) are cumulative from time of diagnosis. Due to the continued prospective follow-up of the patients, at any given time point the total number of cumulative metastatic lesions per patient may change. As an example, patient #23 underwent three resections (one profiled) followed 15 months later by a 4th site of progression that underwent radiotherapy. All sites of metastasis, outside of the CNS, were treated as noted. All intracranial disease was treated with specific doses defined by prospective cooperative group trials. Radiosurgery (SRS) doses were at doses of 15 Gy for 3–4 cm lesions, 18 Gy for 2–3 cm lesions, and 20 Gy for lesions <2 cm in maximum diameter based on Radiation Trials Oncology Group (RTOG) 9005 criteria(i). Abbreviations: For Sample ID, leading Ol = oligometastatic progression or not progressing, Pol = polymetastatic progression; HNSCC = Head and neck squamous cell carcinoma, NSCLC = non small cell lung cancer, Met = sample of metastatic site, # = cumulative count of.
(PDF)

Table S2 **Description of patient characteristics for primary tissue samples ordered by patient ID.** The primary tumor was treated with curative intent and controlled (i.e., no clinical evidence of disease) before the development of metastatic disease in all but four patients, who each had synchronous presentations. Number of metastasis(es) are recorded as cumulative numbers since discovery of primary at the time of “radiation” or of “tissue sampling”. Time to metastasis(es) defined as time to development of metastasis(es) after primary cancer diagnosis. Regional nodal metastasis(es) are not included in this study and all nodal sites listed represent distant metastases. Metastasis(es) needed to be visible on CT or MRI at the time of radiotherapy. The total number of metastasis(es) was limited to ≤5 at the onset of the initial evaluation for treatment. During the follow-up period, patients who remained classified with the oligometastatic state demonstrated a cumulative number of metastasis(es) from 1 to 5 and did not have pericardial, pleural, cerebrospinal, or ascitic fluid. All reported count of metastasis(es) are cumulative from time of diagnosis. Abbreviations: HNSCC = Head and neck squamous cell carcinoma; Ol = oligometastatic progression or not progressing; Pol = polymetastatic progression; Pr = sample of primary tumor, # = cumulative count of.
(PDF)

Table S3 **Characteristics of patients with oligometastatic and polymetastatic progression in metastasis(es) samples.** No patient received chemotherapy concurrently with the radiation therapy. Adjuvant chemotherapy was initiated following RT only for patients showed progression. **Legend**: two-tailed Student t-test (t-test), two-tailed Fisher's Exact Test (FET), non-parametric Mann Whitney Test (MWT), logrank survival test (Logrank), NSCLC = non small cell lung cancer, SCLC = small cell lung cancer; α = 1 brain and 1 lung metastasis from same patient, ∞ = 1 omental and 1 small bowel metastasis from same patient, * = statistically significant.
(PDF)

Table S4 **Characteristics of patients with oligometastatic and polymetastatic progression in primary tumor samples.** No patient received chemotherapy concurrently with the radiation therapy. Adjuvant chemotherapy was initiated following RT only for patients showed progression. **Legend**: two-tailed Student t-test (t-test), two-tailed Fisher's Exact Test (FET), non-parametric Mann Whitney Test (MWT), logrank survival test (Logrank), NSCLC = non small cell lung cancer, SCLC = small cell lung cancer; α = 1 brain and 1 lung metastasis from same patient, ∞ = 1 omental and 1 small bowel metastasis from same patient, * = statistically significant.
(PDF)

Table S5 **Patients and treatment characteristics.** No patient received chemotherapy concurrently with the radiation therapy. Adjuvant chemotherapy was initiated following RT only for patients showed progression. Specifically, 20 of the 34 patients that showed disease progression received adjuvant chemotherapy after RT, among which 9 patients received adjuvant chemotherapy within 6 months of radiation. The rest 14 out of the 34 patients did not receive any additional systemic therapy after RT.
(PDF)

Methods S1 **Supplementary methods.**
(PDF)
