# Supplementary material for: MicroRNA Expression Characterizes Oligometastasis(es)
Source: PLoS One. 2011 Dec 13;6(12):e28650. doi: 10.1371/journal.pone.0028650 (PMC3236765; doi:10.1371/journal.pone.0028650)
Supplement: Figure S1 — Unsupervised hierarchical clustering of primary tumors using the 344 microRNAs filtered from TaqMan miRNA card-A (Methods). Red, black and green represent threshold cycle values above, at or below mean level across all samples. As expected, primary samples were clustered according to the tissue origin and sampling site rather than their oligo or polymetastases classifier. Abbreviations for sampling site: Col = Colon; HNC = Head and Neck carcinoma; Ren = Renal; Lu = Lung; Bre = Breast; Bla = Bladder; Sar = Sarcoma; Liv = Liver; Rec = Rectum; Bow = Small bowel; Che = Chest; Ova = Ovarian; Par = Parotid; Thy = Thymus. (PDF) [file pone.0028650.s001.pdf]

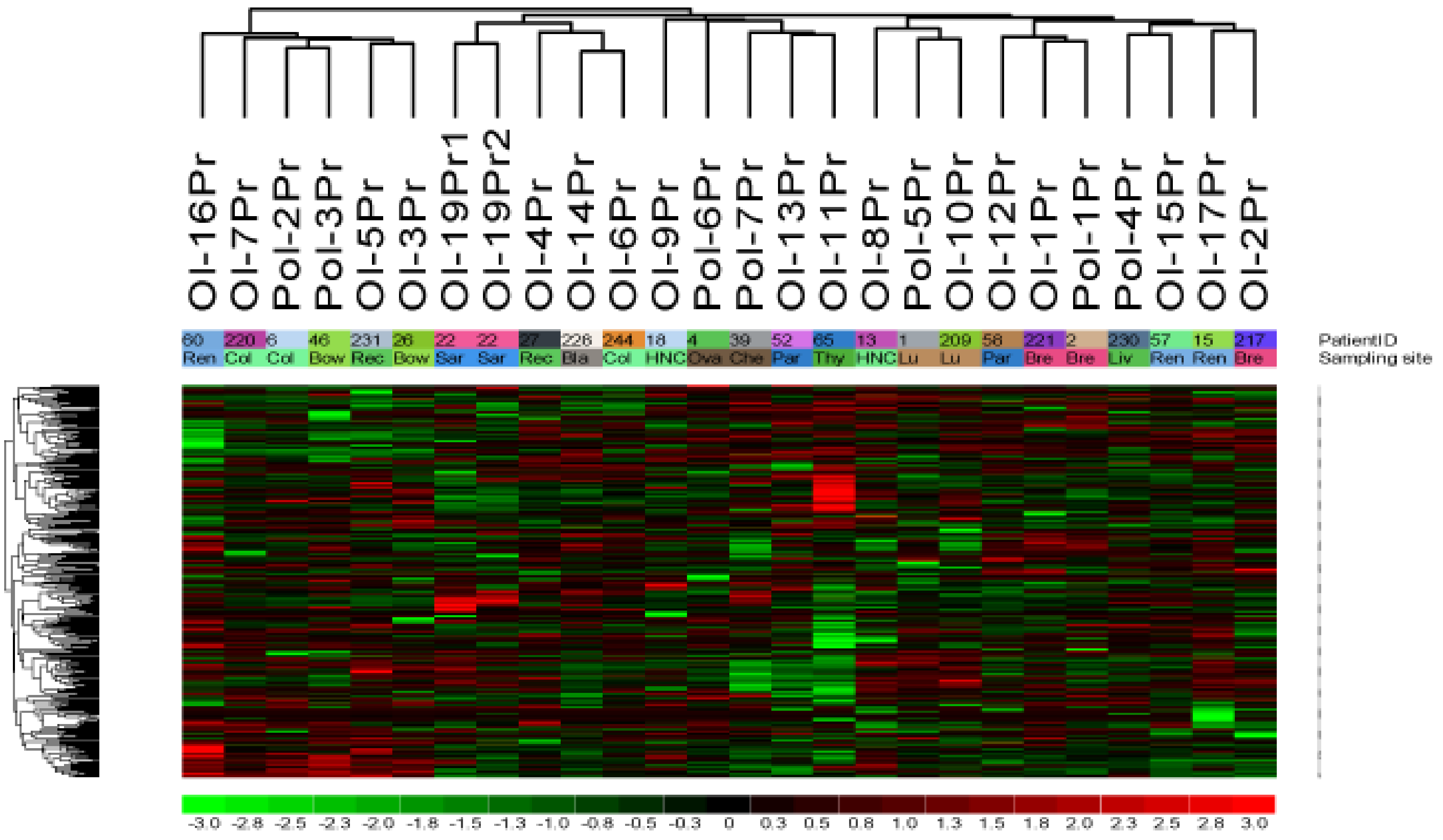

Supplementary Figure S1. **Unsupervised hierarchical clustering of primary tumors using the 344 microRNAs filtered from TaqMan miRNA card-A (Methods).** Red, black and green represent threshold cycle values above, at or below mean level across all samples. As expected, primary samples were clustered according to the tissue origin and sampling site rather than their oligo or polymetastases classifier. Abbreviations for sampling site: Col =Colon; HNC =Head and Neck carcinoma; Ren = Renal; Lu = Lung; Bre = Breast; Bla = Bladder; Sar = Sarcoma; Liv = Liver; Rec = Rectum; Bow = Small bowel; Che = Chest; Ova = Ovarian; Par = Parotid; Thy = Thymus.
