# Supplementary material for: MicroRNA Expression Characterizes Oligometastasis(es)
Source: PLoS One. 2011 Dec 13;6(12):e28650. doi: 10.1371/journal.pone.0028650 (PMC3236765; doi:10.1371/journal.pone.0028650)
Supplement: Figure S2 — Verification of the phenotypic stability of the seven arrayed 2nd generation cell lines via 3rd round of animal modeling. 2×10^6^ purified lung derivative cell lines established from lungs of mice described in Figure 3 and for which the expression was determined ( Fig. 4 ), were injected via tail-vein of 39 NCI female athymic mice (3 oligometastatic L1 and 4 polymetastatic L1Mic cell lines). Animals developing macroscopic observable metastases were sacrificed at the time of this finding. The rest of the animals were sacrificed at 12-weeks post tumor cell injection. Necropsy was performed to score macroscopic metastatic lesions and lungs were harvested and paraffin embedded for histological characterization. While the histology and clinical data reported in Figure 3 refers to the cell lines extracted from lungs at generation two and arrayed, the data reported in this Figure S3 pertain to animals injected with this second generation of cell lines (third round of animal modeling). In mice, the polymetastases MDA-MB-435-GFP-L1Mic cells lines produced more aggressive metastatic progression than the oligometastases MDA-MB-435-GFP-L1 ones in this third animal passage (odds ratio at week 12 = 5.6; P = 0.015; one-tailed FET). (PDF) [file pone.0028650.s002.pdf]

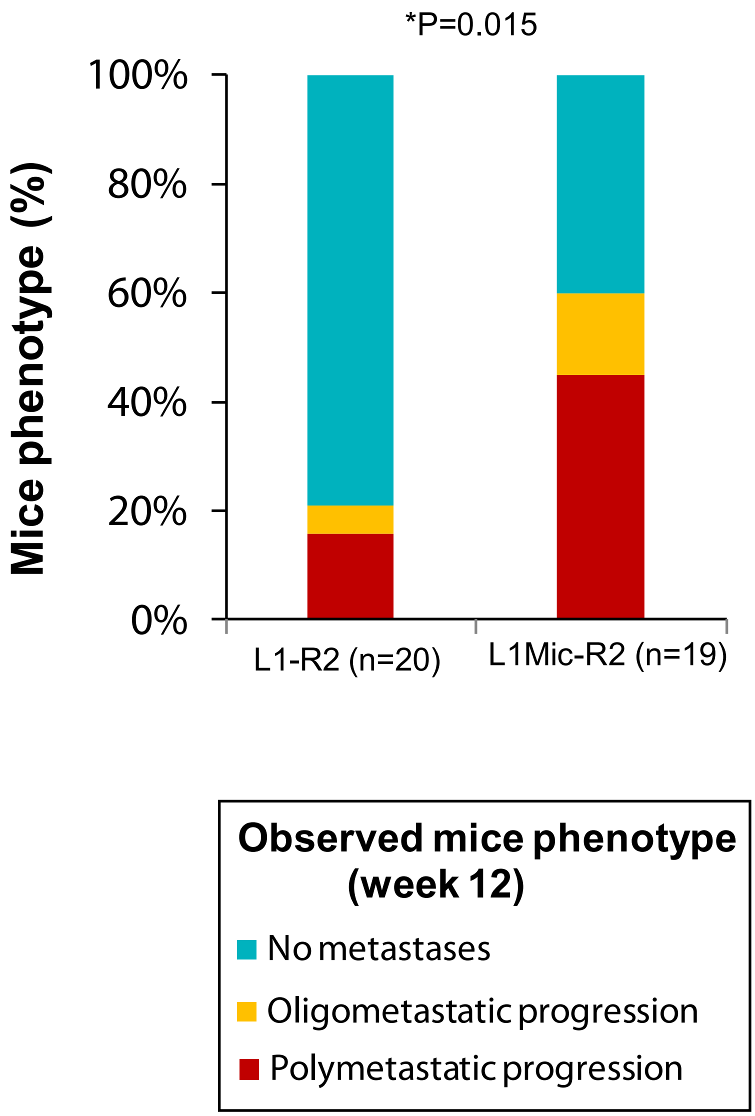

Supplementary Figure S2. Verification of the phenotypic stability of the seven arrayed 2nd generation cell lines via 3rd round of animal modeling. $2x10^6$ purified lung derivative cell lines established from lungs of mice described in **Figure 3** and for which the expression was determined (**Fig. 4**), were injected via tail-vein of 39 NCI female athymic mice (3 oligometastatic L1 and 4 polymetastatic L1Mic cell lines). Animals developing macroscopic observable metastases were sacrificed at the time of this finding. The rest of the animals were sacrificed at 12-weeks post tumor cell injection. Necropsy was performed to score macroscopic metastatic lesions and lungs were harvested and paraffin embedded for histological characterization. While the histology and clinical data reported in **Figure 3** refers to the cell lines extracted from lungs at generation two and arrayed, the data reported in this **Supplementary Figure S3** pertain to animals injected with this second generation of cell lines (third round of animal modeling). In mice, the polymetastases MDA-MB-435-GFP-L1Mic cells lines produced more aggressive metastatic progression than the oligometastases MDA-MB-435-GFP-L1 ones in this third animal passage (odds ratio at week 12 = 5.6; P = 0.015; one-tailed FET).
