# Supplementary material for: MicroRNA Expression Characterizes Oligometastasis(es)
Source: PLoS One. 2011 Dec 13;6(12):e28650. doi: 10.1371/journal.pone.0028650 (PMC3236765; doi:10.1371/journal.pone.0028650)
Supplement: Figure S3 — Quality of microRNA measurement in each human samples. As a control of microRNA quality measure, the number of detectable microRNAs per sample was plotted using the Bioconductor package HTqPCR. Array ID 5a, 15c, and 49b are excluded from the current study because of their excessive number of undetectable microRNAs. Further experiment by PCR of two genes validated the RNA. (PDF) [file pone.0028650.s003.pdf]

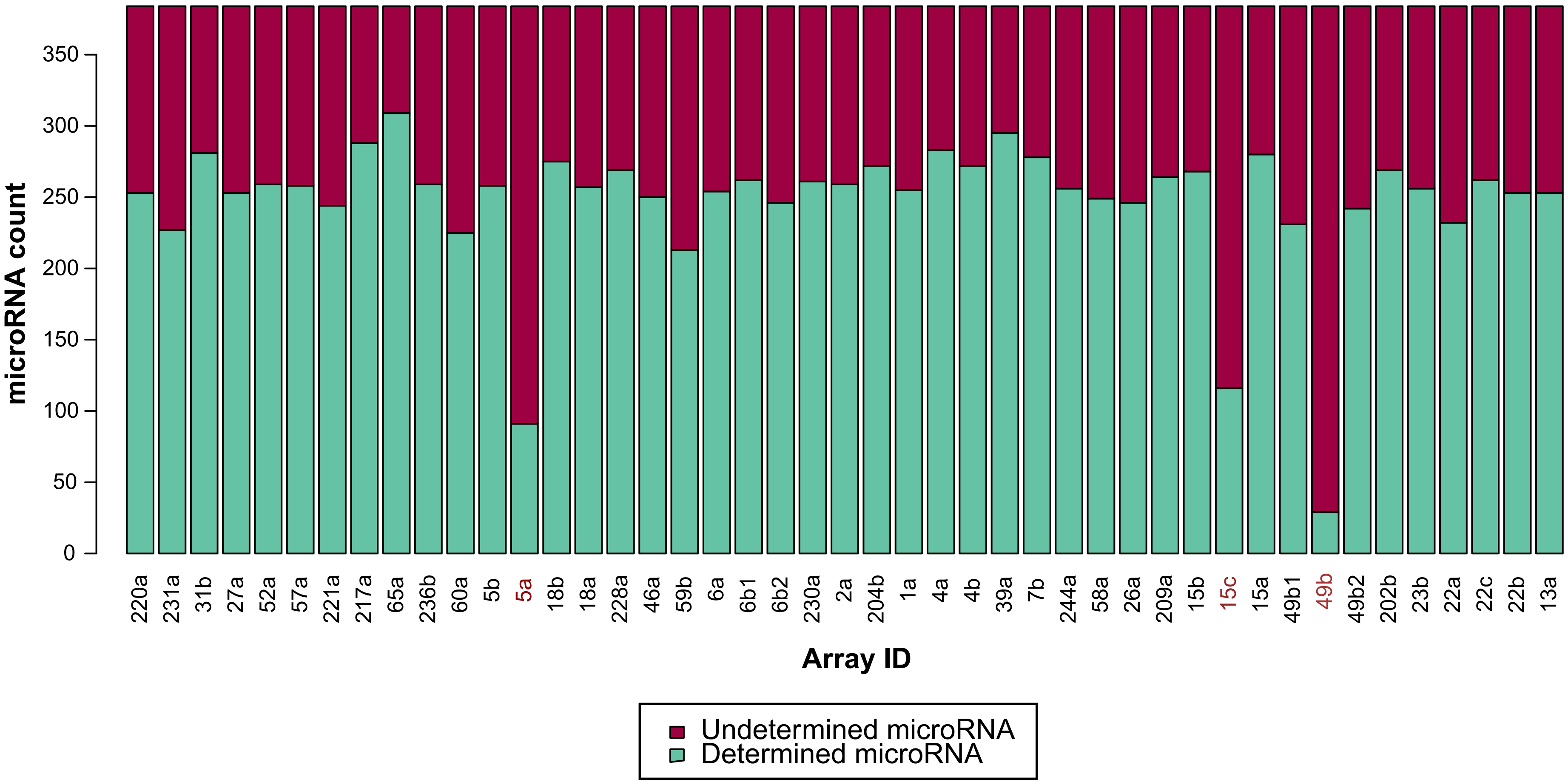

Supplement Figure S3. Quality of microRNA measurement in each human samples.

As a control of microRNA quality measure, the number of detectable microRNAs per sample was plotted using the Bioconductor package HTqPCR. Array ID 5a, 15c, and 49b are excluded from the current study because of their excessive number of undetectable microRNAs. Further experiment by PCR of two genes validated the RNA
