# Supplementary material for: MicroRNA Expression Characterizes Oligometastasis(es)
Source: PLoS One. 2011 Dec 13;6(12):e28650. doi: 10.1371/journal.pone.0028650 (PMC3236765; doi:10.1371/journal.pone.0028650)
Supplement: Figure S4 — The sources of individual samples, each representing a separate lesion is shown. The * represents a single sample excluded because of excessive undetected microRNAs. (PDF) [file pone.0028650.s004.pdf]

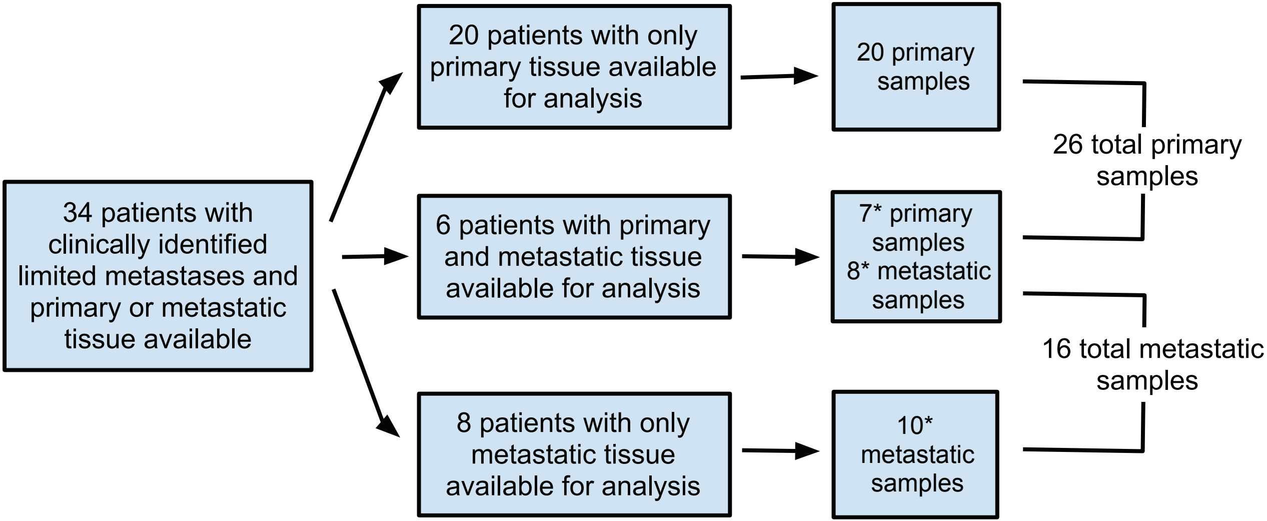

Supplemental Figure S4. The sources of individual samples, each representing a separate lesion is shown. The * represents a single sample excluded because of excessive undetected microRNAs.
