# Supplementary figures and images for: MicroRNA Expression Characterizes Oligometastasis(es)
Source: PLoS One. 2011 Dec 13;6(12):e28650. doi: 10.1371/journal.pone.0028650 (PMC3236765; doi:10.1371/journal.pone.0028650)

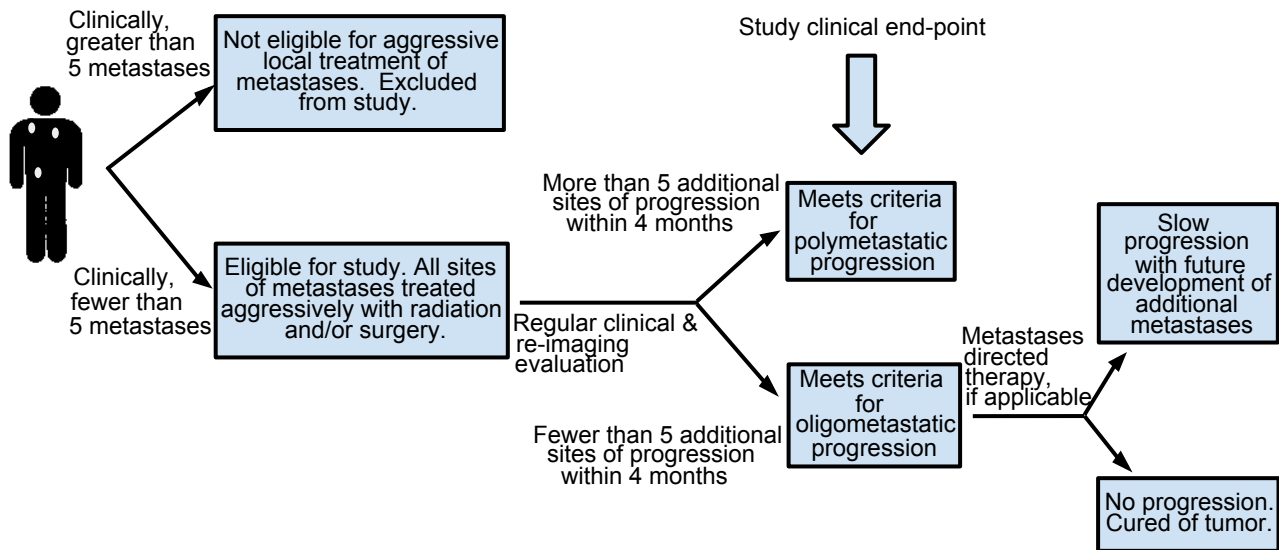

Supplementary Figure S5. Definitions of oligo- and poly- metastatic progression.

Supplement: Figure S5 — Definitions of oligo- and poly- metastatic progression. (PDF) [file pone.0028650.s005.pdf]
