# Supplementary material for: MicroRNA Expression Characterizes Oligometastasis(es)
Source: PLoS One. 2011 Dec 13;6(12):e28650. doi: 10.1371/journal.pone.0028650 (PMC3236765; doi:10.1371/journal.pone.0028650)
Supplement: Table S1 — Description of patient characteristics for the metastatic samples ordered by patient ID. Number of metastasis(es) are listed as cumulative numbers since discovery of primary at the time of “radiation” or of “tissue sampling”. Time to metastasis(es) is defined as time to development of metastasis(es) after primary cancer diagnosis. Regional nodal metastasis(es) are not included in this study and all nodal sites listed represent distant metastases(‡). Metastasis(es) needed to be visible on CT or MRI at the time of radiotherapy. The total number of metastasis(es) was limited to ≤5 at the onset of the initial evaluation for treatment. During the follow-up period, patients who remained classified with the oligometastatic state demonstrated a cumulative number of metastasis(es) from 1 to 5 and did not have pericardial, pleural, cerebrospinal, or ascitic fluid. All reported count of metastasis(es) are cumulative from time of diagnosis. Due to the continued prospective follow-up of the patients, at any given time point the total number of cumulative metastatic lesions per patient may change. As an example, patient #23 underwent three resections (one profiled) followed 15 months later by a 4th site of progression that underwent radiotherapy. All sites of metastasis, outside of the CNS, were treated as noted. All intracranial disease was treated with specific doses defined by prospective cooperative group trials. Radiosurgery (SRS) doses were at doses of 15 Gy for 3–4 cm lesions, 18 Gy for 2–3 cm lesions, and 20 Gy for lesions <2 cm in maximum diameter based on Radiation Trials Oncology Group (RTOG) 9005 criteria(i). Abbreviations: For Sample ID, leading Ol = oligometastatic progression or not progressing, Pol = polymetastatic progression; HNSCC = Head and neck squamous cell carcinoma, NSCLC = non small cell lung cancer, Met = sample of metastatic site, # = cumulative count of. (PDF) [file pone.0028650.s006.pdf]

Supplementary Table S1: Description of patient characteristics for the <u>metastatic</u> samples ordered by patient ID. Number of metastasis(es) are listed as cumulative numbers since discovery of primary at the time of “radiation” or of “tissue sampling”. Time to metastasis(es) is defined as time to development of metastasis(es) after primary cancer diagnosis. Regional nodal metastasis(es) are not included in this study and all nodal sites listed represent distant metastases(‡). Metastasis(es) needed to be visible on CT or MRI at the time of radiotherapy. The total number of metastasis(es) was limited to ≤5 at the onset of the initial evaluation for treatment. During the follow-up period, patients who remained classified with the oligometastatic state demonstrated a cumulative number of metastasis(es) from 1 to 5 and did not have pericardial, pleural, cerebrospinal, or ascitic fluid. All reported count of metastasis(es) are cumulative from time of diagnosis. Due to the continued prospective follow-up of the patients, at any given time point the total number of cumulative metastatic lesions per patient may change. As an example, patient #23 underwent three resections (one profiled) followed 15 months later by a 4th site of progression that underwent radiotherapy. All sites of metastasis, outside of the CNS, were treated as noted. All intracranial disease was treated with specific doses defined by prospective cooperative group trials. Radiosurgery (SRS) doses were at doses of 15Gy for 3-4 cm lesions, 18Gy for 2-3cm lesions, and 20 Gy for lesions <2cm in maximum diameter based on Radiation Trials Oncology Group (RTOG) 9005 criteria[(i)]**.** Abbreviations: For Sample ID, leading Ol = oligometastatic progression or not progressing, Pol = polymetastatic progression; HNSCC = Head and neck squamous cell carcinoma, NSCLC = non small cell lung cancer, Met = sample of metastatic site, # = cumulative count of.

Patient ID	Sample ID	Primary type	Histology	Metastasis(es) location	Gender	Age	Time to Meta-stasis(es) (months)	Meta-stasis(es) at Radiation§ (#)	Metastasis(es) at Metastatic Tissue Sampling§ (#)	Time to last imaging follow-up of "Ol" $^{\Delta}$	Total # of new metastasis(es) within 4 months of 1st metastatic progression after radiotherapy	Alive	Survival (Months)
31	Ol-2Met	Lung	Adenocarcinoma	Adrenal	Male	49.4	0†	2	1	39.3	1	Yes	39.8
*204	Pol-4Met	Lung	NSCLC	Adrenal	Male	58.7	7.9	1	2	n/a	ascites (Pol)	No	18.2
49	Ol-8Met3	Renal	Clear cell	Brain	Female	47.1	48	2	3	23	1	Yes	25.8
59	Pol-2Met	<<<Colon> >>	Adenocarcinoma	Brain	Female	60.3	0.3†	5	9	n/a	9[(ii)]	No	17.9
*202	Ol-3Met	Lung	NSCLC	Chest wall	Female	77.2	6.7	1	1	39	5	Yes	39
5	Ol-4Met	Sarcoma	Fibrosarcoma	Lung	Male	34.2	42.6	3	1	17.8	2	No	18.2
18	Ol-1Met	HNSCC	Squamous cell carcinoma	Lung	Female	69.4	14.2	2	1	10.2	0	No	10.2
49	Ol-8Met1	Renal	Clear cell	Lung	Female	47.1	48	2	1	23	1	Yes	25.8
7	Pol-3Met	Lung	Small cell carcinoma	Lymph node‡	Female	67.4	3.3	1	1	n/a	10	No	12.6
15	Ol-6Met1	Renal	Chromophobe	Lymph node‡	Male	58.9	14.2	3	1	21.7	1	Yes	33.6
23	Ol-7Met	Renal	Papillary adenocarcinoma	Lymph node‡	Male	62	10.2	4	3	37.2	2	Yes	42.5
*236	Ol-5Met	Renal	Papillary renal cell carcinoma	Lymph node‡	Male	80.1	27	3	1	6.9	0	Yes	6.9
6	Pol-1Met1	<<Colon> >	Adenocarcinoma	Omentum	Female	55.8	1.9	5	5	n/a	>10	No	11.5
4	Pol-5Met	Ovarian	Papillary serous carcinoma	Peritoneal implant	Female	70.2	0.0†	1	1	n/a	Peritoneal (Pol)	No	29.5
6	Pol-1Met2	<<Colon> >	Adenocarcinoma	Small bowel	Female	55.8	1.9	5	5	n/a	>10	No	11.5
22	Ol-9Met	Sarcoma	Malignant Fibrohistiocytic	Spleen	Female	55.9	57	4	1	2.5^	3	Yes	29.2

^ This patient was followed up 29.2 months by the research nurse and reports being clinically stable and off chemotherapy with no planned imaging, however last imaging occurred at The U of C at time 2.5 months, which was used conservatively in the calculations of **Supplementary Tables S4-5**. * Patient data collected retrospectively; †Patient #4, #31 and #59 presented with synchronous metastases. The primary tumor was treated with curative surgical intent and locally controlled over follow up. **§**The total cumulative number of metastatic lesions, per patient, did not exceed five during the initial evaluation of the patient. Since multiple metastases could exist in each patient, the total number of metastatic lesions sampled was between one and two. Metastatic tumor tissue for sampling was obtained prior to chemotherapy in all patients from the location deemed by the multidisciplinary team least likely to cause morbidity/complication. $^{\Delta}$ The polymetastatic time to progression is defined by the interval from the presentation of the first site of metastasis until the cumulative number exceeds 5 (>5). **(i)** Shaw E, et al. Int J Radiat Oncol Biol Phys. 1996;34(3):647-54. (ii) This patient had 9 metastases 4.3 months post radiotherapy and was considered Pol as no radiologic assessment was performed between the 9th week to the 17th week post radiation.
