# Supplementary material for: MicroRNA Expression Characterizes Oligometastasis(es)
Source: PLoS One. 2011 Dec 13;6(12):e28650. doi: 10.1371/journal.pone.0028650 (PMC3236765; doi:10.1371/journal.pone.0028650)
Supplement: Table S2 — Description of patient characteristics for primary tissue samples ordered by patient ID. The primary tumor was treated with curative intent and controlled (i.e., no clinical evidence of disease) before the development of metastatic disease in all but four patients, who each had synchronous presentations. Number of metastasis(es) are recorded as cumulative numbers since discovery of primary at the time of “radiation” or of “tissue sampling”. Time to metastasis(es) defined as time to development of metastasis(es) after primary cancer diagnosis. Regional nodal metastasis(es) are not included in this study and all nodal sites listed represent distant metastases. Metastasis(es) needed to be visible on CT or MRI at the time of radiotherapy. The total number of metastasis(es) was limited to ≤5 at the onset of the initial evaluation for treatment. During the follow-up period, patients who remained classified with the oligometastatic state demonstrated a cumulative number of metastasis(es) from 1 to 5 and did not have pericardial, pleural, cerebrospinal, or ascitic fluid. All reported count of metastasis(es) are cumulative from time of diagnosis. Abbreviations: HNSCC = Head and neck squamous cell carcinoma; Ol = oligometastatic progression or not progressing; Pol = polymetastatic progression; Pr = sample of primary tumor, # = cumulative count of. (PDF) [file pone.0028650.s007.pdf]

Supplementary Table S2: Description of patient characteristics for primary tissue samples ordered by patient ID. The primary tumor was treated with curative intent and controlled (i.e., no clinical evidence of disease) before the development of metastatic disease in all but four patients, who each had synchronous presentations. Number of metastasis(es) are recorded as cumulative numbers since discovery of primary at the time of "radiation" or of "tissue sampling". Time to metastasis(es) defined as time to development of metastasis(es) after primary cancer diagnosis. Regional nodal metastasis(es) are not included in this study and all nodal sites listed represent distant metastases. Metastasis(es) needed to be visible on CT or MRI at the time of radiotherapy. The total number of metastasis(es) was limited to ≤5 at the onset of the initial evaluation for treatment. During the follow-up period, patients who remained classified with the oligometastatic state demonstrated a cumulative number of metastasis(es) from 1 to 5 and did not have pericardial, pleural, cerebrospinal, or ascitic fluid. All reported count of metastasis(es) are cumulative from time of diagnosis. Abbreviations: HNSCC = Head and neck squamous cell carcinoma; Ol = oligometastatic progression or not progressing; Pol = polymetastatic progression; Pr = sample of primary tumor, #=cumulative count of.

Patient ID	Sample ID	Primary type	Primary Histology	Gender	Time to meta-stasis(es) (months) (i)	Age (Yrs)	Metas-tasis(es) at Radiation (#)	Meta-stasis(es) at time of primary tissue sampling§ (#)	Time to last imaging follow-up of "Ol" Δ	Total # of new metastasis(es) within 4 months of 1st metastatic progression after radiotherapy	Alive	Survival Months
1	Pol-5Pr	Lung	SCC	Female	16.6	66.1	5	0	n/a	>10	No	10.5
2	Pol-1Pr	Breast	Infiltrating ductal carcinoma	Female	49.6	55.1	1	0	n/a	pleural (Pol)	No	27.4
4	Pol-6Pr	Ovarian	Papillary serous carcinoma	Female	0†	70.2	1	1	n/a	peritoneal	No	29.5
6	Pol-2Pr	Colorectal	Adenocarcinoma	Female	1.9	55.8	5	5	n/a	>10	No	11.5
13	Ol-8Pr	HNSCC	SCC	Male	6.6	64.4	2	0	14.8	1	No	14.9
15	Ol-17Pr	Renal	Chromophobe	Male	14.2	58.9	3	0	21.7	1	Yes	33.6
18	Ol-9Pr	HNSCC	SCC	Female	14.2	69.4	2	0	10.2	0	No	10.2
22	Ol-19Pr1	Sarcoma	Malignant Fibrohistiocytic	Female	57.0	55.9	4	0	2.5^	3	Yes	29.2
22	Ol-19Pr2	Sarcoma	Malignant Fibrohistiocytic	Female	57.0	55.9	4	0	2.5^	3	Yes	29.2
26	Ol-3Pr	Small Bowel	Adenocarcinoma	Male	115.1	62.1	1	0	39.5	3	Yes	43.3
27	Ol-4Pr	Colorectal	Adenocarcinoma	Female	2.3	76.9	2	0	41.5	0	Yes	41.5
39	Pol-7Pr	Sarcoma	Ewings sarcoma	Male	10.1	64.4	1	0	n/a	7	No	11.9
46	Pol-3Pr	Small Bowel	Adenocarcinoma	Male	11.1	53.8	1	0	n/a	ascites (Pol)	No	8.4
52	Ol-13Pr	Parotid	Adenoid cystic	Female	64.3	66.7	3	0	24.1	0	Yes	24.1
57	Ol-15Pr	Renal	Clear cell	Female	42.3	56.3	1	0	13.1	0	Yes	13.1
58	Ol-12Pr	Parotid	Adenocarcinoma	Male	44.8	75.8	1	0	15.2	3	Yes	15.4
60	Ol-16Pr	Renal	Renal cell carcinoma	Female	302.6	70.1	4	1	15.6	0	Yes	18.1
65	Ol-11Pr	Thymus	Large cell neuroendocrine	Male	68.9	48.7	1	0	14.9	0	Yes	14.9
*209	Ol-10Pr	Lung	Adenosquamous	Female	3.7	57.3	2	0	16.1	1	No	17.4
*217	Ol-2Pr	Breast	Infiltrating ductal carcinoma	Female	52.4	91.2	1	0	15.6	0	Yes	15.6
*220	Ol-7Pr	Colorectal	Adenocarcinoma	Female	0	65.3	3	1	14.6	1	Yes	16.9
*221	Ol-1Pr	Breast	Infiltrating ductal carcinoma	Female	126.5	50.2	1	0	18.8	0	Yes	19.0
*228	Ol-14Pr	Bladder	Urothelial Carcinoma	Male	7.5	65.8	1	0	6.8	1	Yes	8.7
*230	Pol-4Pr	Liver	Hepatocellular carcinoma	Male	39.1	62.3	1	0	n/a	6	Yes	11.1
*231	Ol-5Pr	Colorectal	Adenocarcinoma	Male	63.4	70.1	4	0	5.7	0	Yes	5.7
*244	Ol-6Pr	Colorectal	Adenocarcinoma	Male	49.3	70.1	1	0	10.6	3	Yes	10.6

* Patient data collected retrospectively; ^ This patient was followed up 29.2 months by the research nurse and reports being clinically stable and off chemotherapy with no planned imaging, however last imaging occurred at The U of C at time 2.5 months. † Patient #4 presented with synchronous metastases. The primary tumor was treated with curative surgical intent and locally controlled over follow up. §The total cumulative number of metastatic lesions, per patient, did not exceed five during the initial evaluation of the patient. Since multiple metastases could exist in each patient, the total number of metastatic lesions sampled was between one and two. Metastatic tumor tissue for sampling was obtained prior to chemotherapy in all patients from the location deemed by the multidisciplinary team least likely to cause morbidity/complication. Δ The polymetastatic time to progression is defined by the interval from the presentation of the first site of metastasis until the cumulative number exceeds 5 (>5). (i) Four patients were diagnosed with metastatic disease within one month of the primary tumor diagnosis. For these patients the primary tumor was addressed with curative l surgical intent and controlled with follow up as well.
