# Supplementary material for: MicroRNA Expression Characterizes Oligometastasis(es)
Source: PLoS One. 2011 Dec 13;6(12):e28650. doi: 10.1371/journal.pone.0028650 (PMC3236765; doi:10.1371/journal.pone.0028650)
Supplement: Table S3 — Characteristics of patients with oligometastatic and polymetastatic progression in metastasis(es) samples. No patient received chemotherapy concurrently with the radiation therapy. Adjuvant chemotherapy was initiated following RT only for patients showed progression. Legend: two-tailed Student t-test (t-test), two-tailed Fisher's Exact Test (FET), non-parametric Mann Whitney Test (MWT), logrank survival test (Logrank), NSCLC = non small cell lung cancer, SCLC = small cell lung cancer; α = 1 brain and 1 lung metastasis from same patient, ∞ = 1 omental and 1 small bowel metastasis from same patient, * = statistically significant. (PDF) [file pone.0028650.s008.pdf]

Supplementary Tables S3. Characteristics of patients with oligometastatic and polymetastatic progression in metastasis(es) samples.

No patient received chemotherapy concurrently with the radiation therapy. Adjuvant chemotherapy was initiated following RT only for patients showed progression. **Legend**: two-tailed Student t-test (t-test), two-tailed Fisher's Exact Test (FET), non-parametric Mann Whitney Test (MWT), logrank survival test (Logrank), NSCLC = non small cell lung cancer, SCLC = small cell lung cancer; α=1 brain and 1 lung metastasis from same patient, ∞ = 1 omental and 1 small bowel metastasis from same patient, *=statistically significant.

**Patients** (#)	**All** (N=14)	**Oligometastatic Progression** (N=9)	**Polymetastatic Progression** (N=5)	**p-value**
**Median Age** (years)	59.6	58.9	60.3	0.66 (t-test)
Range:	(34.2-80.1)	(34.2-80.1)	(55.8-70.2)	
**Sex**				0.30 (FET)
Female (%)	8 (57%)	4 (44%)	4 (80%)	
Male (%)	6 (43%)	5 (56%)	1 (20%)	
**Primary tumor type**	14	9	5	
Colorectal	2	0	2	
Head and Neck	1	1	0	
Lung, NSCLC	3	2	1	
Lung, SCLC	1	0	1	
Ovarian	1	0	1	
Renal	4	4	0	
Sarcoma	2	2	0	
**Metastatic Tissue Origin**	16	10	6	
Adrenal metastasis	2	1	1	
Brain α	2	1	1	
Chest wall metastasis	1	1	0	
Lung metastasis α	3	3	0	
Lymph node metastasis	4	3	1	
Peritoneal/omental metastasis∞	2	0	2	
Small bowel metastasis∞	1	0	1	
Spleen Metastasis	1	1	0	
**Median # prior and current metastasis(es) at radiation** (Range)	2.5 (1-5)	3 (1-4)	1 (1-5)	0.70 (MWT)
**Median # of metastasis(es) at tissue sampling** (Range)	1 (1-9)	1 (1-3)	2 (1-9)	0.24 (MWT)
**Survival:** (alive throughout follow-up/ total per group)	7 / 14	7 / 9	0 / 5	**0.021*** (FET)
**Median follow-up:** (months)	22.0	29.2	17.9	**0.014***
Range:	(6.9-42.5)	(6.9-42.5)	(11.5-29.5)	(Logrank)
