# Supplementary material for: MicroRNA Expression Characterizes Oligometastasis(es)
Source: PLoS One. 2011 Dec 13;6(12):e28650. doi: 10.1371/journal.pone.0028650 (PMC3236765; doi:10.1371/journal.pone.0028650)
Supplement: Table S5 — Patients and treatment characteristics. No patient received chemotherapy concurrently with the radiation therapy. Adjuvant chemotherapy was initiated following RT only for patients showed progression. Specifically, 20 of the 34 patients that showed disease progression received adjuvant chemotherapy after RT, among which 9 patients received adjuvant chemotherapy within 6 months of radiation. The rest 14 out of the 34 patients did not receive any additional systemic therapy after RT. (PDF) [file pone.0028650.s010.pdf]

Supplemental Table S5: Patients and treatment characteristics.

No patient received chemotherapy concurrently with the radiation therapy. Adjuvant chemotherapy was initiated following RT only for patients showed progression. Specifically, 20 of the 34 patients that showed disease progression received adjuvant chemotherapy after RT, among which 9 patients received adjuvant chemotherapy within 6 months of radiation. The rest 14 out of the 34 patients did not receive any additional systemic therapy after RT.

Characteristic	No. of patients
No. of Patients	34 (100%)
Median Age (range)	63.3 (34.2-91.2)
**Radiographic Staging Modality**	
PET/CT	18 (53%)
CT chest/ab/pelvis	7 (21%)
CT chest/ab/pelvis & bone scan	6 (18%)
MRI brain, CT chest/ab/pelvis	1 (3%)
MRI brain, PET scan	1 (3%)
MRI pelvis, bone scan	1 (3%)
**Chemotherapy**	
Prior systemic chemotherapy	27 (80%)
Post HIGRT chemotherapy	20 (59%)
Within 6 mo	9 (26%)
6-12 months	5 (15%)
Greater than 12 months	6 (18%)
