# Supplementary material for: MicroRNA Expression Characterizes Oligometastasis(es)
Source: PLoS One. 2011 Dec 13;6(12):e28650. doi: 10.1371/journal.pone.0028650 (PMC3236765; doi:10.1371/journal.pone.0028650)
Supplement: Methods S1 — Supplementary methods. (PDF) [file pone.0028650.s011.pdf]

Supplementary Methods.

Summary of the Radiotherapy for Oligometastasis(es). Patients underwent computed tomography (CT) based radiation treatment planning accounting for respiratory induced tumor motion and intravenous and/or oral contrast media as determined by treating physician. The attending radiation oncologist contoured tumors (GTV) using all available clinical, radiographic, and metabolic data. The volume(s) wasexpanded 5-10 mm to account for set-up error. A variety of non-overlapping axial and non-coplanar delivered the optimal radiation distribution while minimizing radiation to surrounding non-involved organs. Normal tissue tolerances were estimated from the available literature when designing the radiation plans[33-35]. Typically, radiation was delivered in three doses (8-16 Gy per dose) for those treated on protocol and in a ten-dose regimen (50 Gy total dose, 5 Gy per dose) for those treated off protocol.

Validation of prioritized oligometastasis(es) vs polymetastases microRNA signatures using independent datasets and ROC curves. PCA and 1st component are calculated in the validation sets using the Pr-miRs and M-miRs microRNA lists (**Methods, Table 1a-b**). The computed first component was then used to generate an ROC (Receiver Operating Characteristic) curve using R "caTools"[31] package of Bioconductor[30] for the validation in human samples (**Fig. 2a-b**). The ROC curve plots the true positive rate against the false positive rate according to different possible thresholds for oligo vs polymetastases determination. An empirical p-value was calculated for the area under the curve of the ROC (AUC) by permutation resampling (**Table 2a-b**). In each permutation, class assignment of oligometastases(es) or polymetastases was sampled without replacement in the validation sets. This simulation was performed 1000 times for metastatic tumor samples using Pr-miRs and likewise for primary tumor samples using M-miRs. For the L1 and L1Mic validation sets, this procedure was performed on the exact 35 possible outcome combinations of samples. We thus generated an empirical distribution of AUCs for separating oligometastatic - from polymetastatic samples using the 17 Pr-miRs and the 29 M-miRs described in **Table 1a-b**, respectively. Scatter plots and non-parametric Mann-Whitney tests were performed using the GraphPad Prism version 4.03 (**Fig. 4a-b**).

REporting recommendations for tumor MARKer prognostic studies (REMARK)[1].

The current study samples and analyses satisfy nine of the twelve REMARK criteria: #1-2, #4-8, and #11[1].

1 McShane LM, Altman DG, Sauerbrei W, Taube SE, Gion M, Clark GM; Statistics Subcommittee of the NCI-EORTC Working Group on Cancer Diagnostics. REporting recommendations for tumor MARKer prognostic studies (REMARK). Nat Clin Pract Oncol. 2005 Aug;2(8):416-22.
